# Sweet Immunity Aspects during Levan Oligosaccharide-Mediated Priming in Rocket against *Botrytis cinerea*

**DOI:** 10.3390/biom12030370

**Published:** 2022-02-25

**Authors:** Maxime Versluys, Wim Van den Ende

**Affiliations:** Laboratory of Molecular Plant Biology and KU Leuven Plant Institute, KU Leuven, Kasteelpark Arenberg 31, 3001 Leuven, Belgium; maxime.versluys@kuleuven.be

**Keywords:** fructan, priming, sweet immunity, *Botrytis cinerea*, sugars, levan oligosaccharide, apoplast

## Abstract

New strategies are required for crop protection against biotic stress. Naturally derived molecules, including carbohydrates such as fructans, can be used in priming or defense stimulation. Rocket (*Eruca sativa*) is an important leafy vegetable and a good source of antioxidants. Here, we tested the efficacy of fructan-induced immunity in the *Botrytis cinerea* pathosystem. Different fructan types of plant and microbial origin were considered and changes in sugar dynamics were analyzed. Immune resistance increased significantly after priming with natural and sulfated levan oligosaccharides (LOS). No clear positive effects were observed for fructo-oligosaccharides (FOS), inulin or branched-type fructans. Only sulfated LOS induced a direct ROS burst, typical for elicitors, while LOS behaved as a genuine priming compound. Total leaf sugar levels increased significantly both after LOS priming and subsequent infection. Intriguingly, apoplastic sugar levels temporarily increased after LOS priming but not after infection. We followed LOS and small soluble sugar dynamics in the apoplast as a function of time and found a temporal peak in small soluble sugar levels. Although similar dynamics were also found with inulin-type FOS, increased Glc and FOS levels may benefit *B. cinerea.* During LOS priming, LOS- and/or Glc-dependent signaling may induce downstream sweet immunity responses.

## 1. Introduction

To sustain food demands for the growing human population, it is important to increase crop yields. Several (a)biotic stressors have a negative impact on crop growth, and these effects are expected to become more frequent due to climate change [1,2]. Chemical control remains the main strategy to decrease biotic stress exposure and effects, although negative effects on environment and health are well known [3]. More sustainable alternatives are steadily implemented in integrated pest management systems, including biological control and immune system priming [4,5,6], the latter increasing the immune response of the plant to future stress exposure. Some priming compounds can also be used as prebiotics to stimulate the positive effects of beneficial micro-organisms [7,8,9]. 

A first tier in the plant immune system involves the recognition of microbial effectors by specific resistance proteins, evoking a strong immune response [10,11]. In the second tier, pattern recognition receptors (PRRs) recognize conserved microbe-associated molecular patterns (MAMPs) [12,13,14], such as flg22 recognition by FLS2 [15,16], or chitin by LYK5 and CERK1 in *Arabidopsis thaliana* [17,18,19]. Chitin is the main component of the fungal cell wall and through action of plant chitinases chitin oligomers of lower degree of polymerization (DP) are produced, which are known to trigger immune responses through binding the above-mentioned receptors [20,21]. Besides the recognition of non-self patterns, plants can also sense and trigger defense response to self-type molecules, including molecules leaked into the apoplast during cellular rupture, or breakdown products of the plant cell wall [22,23]. Since they are under evolutionary control of the plant, these damage-associated molecular patterns (DAMPs) provide an additional advantage and are expected to be important for MAMP-triggered immune induction in the roots [24]. In *A. thaliana*, recognition of pectin degradation fragments (oligogalacturonides, OGs) from plant homogalacturonan and recognition by a WAK1 PRR have been studied in detail [25,26]. Other potential DAMPs, such as cello-oligosaccharides, have been identified lately [27,28].

Reactive oxygen species (ROS) bursts and Ca^2+^ fluxes are early signals that are induced after immune system activation by elicitors or apoplastic osmolytes [29,30,31]. NADPH oxidase RBOHD creates a burst of superoxide in the apoplast, and is a central hub towards downstream responses [32,33], including MAPK (mitogen-activated protein kinase) cascades, which phosphorylate further downstream targets [34,35]. Many of these are transcription factors that regulate gene expression related to defense and phytohormone pathways [36,37]. Salicylic acid (SA), jasmonic acid and ethylene are central phytohormones in defense regulation and the induction of defense responses, such as production of secondary metabolites and pathogenesis-related proteins, or creating physical barriers against pathogen invasion [38,39,40,41]. Constitutive defenses are also of importance. Glucosinolates in Brassicaceae are hydrolyzed by myrosinase enzymes during cellular damage, producing toxic by-products to deter biotic stressors [42,43].

An initial trigger primes the plant in a systemic way, leading to a stronger and faster response to future stresses [44,45]. Recognition of pathogenic or beneficial micro-organisms is also known to trigger an induced resistance, but chemical compounds of biological origin can induce similar effects [46,47,48]. Since priming is considered cost-effective for the plant, it provides an interesting strategy to increase crop disease resistance [49]. Many compounds have been identified as promising priming agents, including amino acids [50], organic acids [51], polyamines [52], γ-aminobutyric acid (GABA) [53] and carbohydrates [54,55,56,57,58,59].

Glucose (Glc), fructose (Fru) and sucrose (Suc) are central in the primary metabolism of the plant, together with invertases, which degrade Suc into Glc and Fru [60,61,62]. Moreover, these small soluble sugars play an important role as signaling compounds in growth and development [63], but also in plant defense regulation (sweet immunity concept) [64]. Exogenous application of different sugars, such as trehalose [65] and Suc [66], can lead to induced resistance. Several studies have also confirmed the induced resistance of plants after treatment with oligosaccharides, such as OGs [67], cellulose oligomers [28] and β-glucans [68]. Thus, the sweet immunity concept extends towards carbohydrates with higher DP [69], and in this regard fructans are gaining interest. They are oligo- and polysaccharides consisting of Fru [70] and found in many bacteria and in ca. 15% of flowering plants. Their synthesis is performed by different types of fructosyltransferases [71,72,73]. Fructans have either β-2,1 (inulin) or β-2,6 (levan) glycosidic bonds. Dicot plants mainly produce inulin, while levan, mixed-type fructans (graminans) and neokestose-based fructans are mostly found in monocots [73]. Plant fructan exohydrolases (FEHs) catalyze the degradation of fructan by hydrolyzing the terminal glycosidic bond [74].

Besides their role in abiotic stress responses [75], several studies have highlighted the potential of fructans as priming agents to counteract biotic stress. Inulin priming improved plant resistance to infection in *A. thaliana* [56], *Lactuca sativa* (lettuce) [8] and other crop species [76,77], while levan improved tolerance of *Malus domestica* (apple) [78]. In *A. thaliana*, improved resistance to *B. cinerea* was especially evident when using low DP levan oligosaccharides (LOS) [56]. Sulfated levan-type fructans were shown to have promising immune-stimulating effects in animals [79]. Levan is produced by many bacteria, while inulin production is limited to a few taxonomic groups [80]. Microbial fructan is characterized by a very high DP, produced outside the cell and incorporated into the extracellular matrix [73]. Therefore, it is possible that they can be sensed by the plant as MAMP [81]. In animals, it was shown that fructans can trigger a direct immune activation through toll-like receptor (TLR) activation [82,83]. Plants do not contain TLR homologues, but may contain other, so far unidentified receptors with similar functions. However, due to the high DP of microbial fructan, limited mobility in the apoplast is envisioned. After partial degradation, LOS will likely be a more effective candidate signaling entity, especially in the context of plant-microbial interaction [84]. Fructan degradation requires action of microbial endo-fructanases, plant apoplastic FEHs, or both. 6-FEHs with levan-hydrolyzing activity have been identified in several non-fructan plants [85,86,87], and an apoplastic localization has been confirmed for a chicory 6-FEH recently [88], further confirming a putative function in degrading microbial levan into LOS, acting as prebiotics and/or signaling compounds in the plant rhizosphere [84,88]. Possibly, similar mechanisms may be at play in the plant phyllosphere as well.

Fructan priming through leaf spraying has been studied in several plant species. Recently, the model plant *A. thaliana* was also considered, showing promising effects for inulin and LOS treatments [56]. In this manuscript, we tested fructan priming in *Eruca sativa* (rocket), a crop species that also belongs to the Brassicaceae family, such as *A. thaliana*. It has become popular as food, often mixed in salads. This species is originally from Mediterranean regions, hence it has a drought tolerant nature, allowing cultivation in conditions where water is scarce [89]. It is a natural source of antioxidants and health-promoting phytochemicals, including glucosinolates and flavonols. Rocket oil also has antimicrobial properties [90]. Although most priming studies focused on one or a few fructan types, we screened a large array of fructans with different linkage type, origin (plant or bacteria) and DP in the *E. sativa-B. cinerea* pathosystem. *B. cinerea* is a widespread pathogenic fungus that can infect over 500 plant species, including many crops [91,92]. This screening will allow us to pinpoint the most promising fructan priming agents, which will be investigated deeper. Good priming efficacy is expected for low DP fructans and especially LOS. Disease resistance, sugar dynamics and defense responses were studied for the most promising fructan species. We investigated how fructan oligosaccharide priming influenced levels of small soluble sugars and starch, as well as invertase activities. Small soluble sugars were also measured in extracts from the apoplast after priming, and the results indicate sugar-based signaling responses. To discern between priming or direct immune-eliciting effects, ROS bursts and activities of some immunity-related enzymes were measured as well. Finally, we investigated whether sprayed fructan oligosaccharides actually reached the apoplast. The observed results are discussed in light of the sweet immunity context.

## 2. Materials and Methods

### 2.1. Biological Material

Seeds of *Eruca sativa* Pronto (Sanac; Wervik, Belgium) were transferred to potting soil in square pots (7 cm × 7 cm × 9 cm). The soil contained 14 kg m^−3^ nitrogen, 16 kg m^−3^ phosphorus and 18 kg m^−3^ potassium. Plants were grown under a 12 h light (22 °C) and 12 h dark (18 °C) cycle with a light intensity of 170 µmol m^−2^ s^−1^ in a Conviron^®^ growth chamber (Berlin, Germany). All plants used for the experiments were 4 to 5 weeks old.

*Botrytis cinerea* strain B05.10 (Prof B. De Coninck; KU Leuven, Leuven, Belgium) spores were plated on PDA (potato dextrose agar, 24 g L^−1^) plates for 14 d in the dark at 21 °C according to Janse van Rensburg et al. [56]. Using sterile 0.0001% Tween-20, spores were harvested from the plate and filtered through glass wool. Spore concentration was measured using a Neubauer hemocytometer and a light microscope. Spores were hydrated overnight at 4 °C. Before infections, spores were inoculated briefly in infection buffer, containing 12 g L^−1^ potato dextrose and 1 × 10^5^ spores mL^−1^, allowing synchronized germination. Infection control (IC) buffer contained 12 g L^−1^ potato dextrose without spores. The obtained spore solution was used to inoculate new PDA plates for subsequent experiments.

For inulinase assays, a piece of the *B. cinerea* agar plate was placed upside down on liquid medium and grown in the dark. Time points were taken by heating liquid medium at 90 °C for 10 min. Samples were partially purified through Dowex^®^ ion exchange resins before loading on a high-performance anion exchange chromatograph with integrated pulsed amperometric detection (HPAEC-IPAD) for analysis. Liquid medium consisted of 1 × Vogels minimal medium [93] without carbon source. HP chicory inulin was added as carbon source. All substrates were used with 5 g L^−1^ end concentration and filter sterilized.

### 2.2. Fructan Priming Compound Preparations

HP chicory inulin and chicory FOS (P95) were purchased from Beneo–Orafti (Tienen, Belgium). The latter is derived from HP inulin treatment with an endo-inulinase. Microbial inulin from *Lactobacillus gasseri* DSM 20243 (Prof M.A. Anwar; National Institute for Biotechnology and Genetic Engineering, Faisalabad, Pakistan) and an *Aspergillus niger* endo-inulinase (Megazyme, Bray, Ireland) were used to produce an inulin FOS fraction, similar to P95, through incubation in NaOAc buffer pH 5.0 at 55 °C. Reactions were heated to 90°C for 5 min and centrifuged at 4 °C and 7800× *g* for 10 min. Supernatant was used for fractionation of the reaction products and purification of the FOS fraction (fraction 4) using stepwise precipitation with different concentrations of acetone. The supernatant of the 80% acetone solution contained the FOS fraction. Acetone was evaporated using a rotary evaporator and the resulting pellet resuspended in ddH_2_O, before being purified through a Dowex^®^ column containing H^+^ and Ac^−^ resins (Sigma-Aldrich, St. Louis, MO, USA). Before lyophilization (LSL Secfroid, Aclens, Switzerland), samples were adjusted to pH 7.0 using sodium bicarbonate.

*Halomonas smyrnensis* AAD6^T^ [94], *Bacillus subtilis* levan (Montana Polysaccharides Corp, Winnsboro, TX, USA) and LOS fractions were produced using a purified endo-levanase from *Bacteroides thetaiotaomicron* BT1760 (Prof T. Visnapuu; University of Tartu, Tartu, Estonia). The enzyme was heterologously expressed in *Escherichia coli* and His tag purified according to Mardo et al. [95]. Reactions were performed at 30 °C in NaOAc buffer pH 5.0. Further fractionation and purification to obtain the LOS fraction followed the methodology described for inulin FOS. The extraction and purification of levan from *Dactylis glomerata* followed a protocol from Maleux and Van den Ende [96]. Briefly, excised leaves were incubated under continuous light in 100 mM Suc to induce levan production. After an initial EtOH extraction, a water extract was filtered and exposed to several liming and carbonation rounds. After Dowex purification, levans were precipitated using 60% acetone and the precipitate was lyophilized.

Graminans were extracted from wheat bran (*Triticum aestivum*) according to Verspreet et al. [97]. After water extraction and subsequent EtOH (66%) precipitation, a lyophilized powdered sample was incubated with an α-galactosidase to degrade raffinose. A fructo-oligosaccharide fraction was then purified using gel filtration chromatography. Agavins from *Agave tequilana* were purchased from Preventy (Capilla de Guadalupe, Mexico). All fructan fractions were validated on a HPAEC-IPAD. If considerable contamination with small soluble sugars was detected, additional purifications were performed using a NH_2_ (amino) SPE sorbent column (VWR, Radnor, PA, USA) and different concentrations of acetonitrile. Fructan samples were loaded onto the column in 80% acetonitrile and washed to remove small soluble sugars. The column was washed with ddH_2_O to elute higher DP fructan.

Sulfated levan (Prof E. Toksoy Öner; Marmara University, Istanbul, Turkey) was produced from *H. smyrnensis* levan according to Erginer et al. [79] with a degree of sulfation of 2.25. Sulfated LOS was produced through incubation with *B. thetaiotaomicron* endo-levanase as described above with small modifications. Only Dowex H^+^ resin was used for final purification. Instead of HPAEC-IPAD, TLC methods were used for validation of hydrolysis reaction products, due to the negative charge of the sulfated levan. A silica gel was used as stationary phase, while a mobile phase consisted of butanol:methanol:H_2_O:acetic acid in a 50:25:20:1 ratio. A phosphoric acid and urea-based staining solution was used for visualization at 100 °C [98].

### 2.3. Leaf Priming, Sampling and Disease Scoring Assay

Rocket plants of 4 to 5 weeks old were randomly separated into the given treatment groups. All plants were well watered the day before performing the treatments. The following treatments were used: H_2_O, untreated control, hexaethylene glycol (HEG), γ-aminobutyric acid (GABA), HP chicory inulin, P95 chicory FOS, *L. gasseri* inulin, *L. gasseri* FOS, *H. smyrnensis* levan, *H. smyrnensis* hydrolysis fraction 1 to 4 (fraction 4 = LOS), *B. subtilis* levan, *B. subtilis* LOS, *D. glomerata* levan, sulfated levan, sulfated LOS, wheat graminans and *A. tequilana* agavin. Tween-20 was used as a surfactant for all preparations at 0.001%. Untreated control plants were not sprayed. HEG (Sigma-Aldrich, St. Louis, MO, USA) was used at 15 mM as an osmotic control. GABA was used as positive control in the low mM range [53]. All fructans were used at 5 g·L^−1^. Source leaves were sprayed adaxially with priming solution until the entire surface was covered homogenously.

Two to three source leaves per plant were detached and subjected to leaf priming. In our initial experiments focusing on the whole fructan array, 72 h leaf priming was used, but when focusing on microbial LOS, 24 h priming appeared sufficient to obtain disease protection. Before further manipulation, leaves were rinsed with ddH_2_O to remove residual priming residue and carefully dried with paper towel [8,56]. Dried leaves were placed onto moist paper towel inside circular petri dishes (Greiner Bio-One, Frickenhausen, Germany). Three droplets of 5 µL infection buffer (or IC) were evenly distributed onto each leaf, avoiding main veins. Plates were closed off with parafilm-sealed lids and incubated at 18 °C in a 12 h light/12 h dark cycle for 72 h. Necrotic lesions were measured together with a 1 cm^2^ reference by taking pictures, followed by analysis using ImageJ 1.5T software (https://imagej.nih.gov/ij/, last accessed on 12 January 2017).

Leaf sampling was performed 24 h after priming or 48 h after spore inoculation, using 6 plants per treatment. Leaf mesophyll was flash-frozen in liquid nitrogen and crushed to a fine homogenous powder. Samples at 24 h after priming were rinsed with ddH_2_O as described above. Residual infection buffer was removed from leaves sampled 48 h after inoculation.

### 2.4. Extraction of Soluble Sugars and Starch

Extraction of soluble sugars followed boiling of samples in ddH_2_O as previously described by Vergauwen et al. [99]. Fifty mg of grinded sample was boiled at 100 °C for 10 min. After centrifugation at 15,000× *g* for 10 min, 200 µL of supernatant was transferred to a Dowex^®^ column containing H^+^ and Ac^−^ resins. The column was washed six times with 200 µL ddH_2_O and the entire flowthrough collected. Samples were centrifuged for 5 min at 15,000× *g* before analysis on HPAEC-IPAD. Glc, Fru, Suc, total hexoses, total sugars and Suc/hexose ratio were analyzed.

Starch extraction followed a protocol adapted from Smith and Zeeman [100]. Fifty mg of sample was washed three times with 80% EtOH. Samples were heated to 82 °C for 5 min, centrifuged for 5 min at 15,000× *g* and supernatant removed. Starch extraction was performed at 100 °C for 10 min in 500 µL ddH_2_O after which samples were again centrifuged. The supernatant was used to set up reactions for starch degradation in 350 mM NaOAc buffer pH 5.0. Reactions contained 10 mg mL^−1^ α-amylase and 10 mg mL^−1^ amyloglucosidase to degrade the starch content in 100 µL of sample overnight at 30 °C. Reactions were halted by boiling samples at 100 °C for 5 min. Enzymes were deactivated before addition to the sample for blank controls. Starch content was expressed in terms of the produced Glc levels from the hydrolysis reactions.

### 2.5. Invertase Activity Assays

Both cell-wall invertase (cwINV) and vacuolar invertase (vINV) activity were measured for all samples according to Tarkowski et al. [8], with minor modifications. One hundred mg grinded sample was added to 500 µL ice-cold extraction buffer containing 50 mM NaOAc pH 5.0, 10 mM NaHSO_3_, 2 mM β-mercaptoethanol, 0.1% polyclar and 100 µM PMSF. After extraction on ice using a micro pestle, samples were centrifuged for 20 min at 20,000× *g* and 4 °C. The resulting pellet contained the cwINV fraction, while vINV activity was measured on the supernatant. The pellet was washed several times with 50 mM NaOAc buffer pH 5.0 through centrifugation (10 min at 20,000× *g* and 4 °C) and pellet resuspension using a micro pestle. In the final resuspension step, 500 µL buffer was added, including 50 mM Suc to start the reaction. After a 30 min incubation time at 30 °C and 600 rpm, samples were heated to 95 °C for 5 min to stop the reaction. Zero min control samples were taken from the reaction and heated immediately. In the supernatant fraction, vINV was further purified through ammonium sulfate (80%) precipitation on ice in two subsequent rounds (centrifugation at 4 °C for 5 min at 20,000× g). The final pellet was resuspended in 50 mM NaOAc buffer pH 5.0. One hundred mM Suc was added, and reactions were incubated at 30 °C for 30 min, before stopping the reactions by heating to 95 °C for 5 min. Zero min control samples were immediately taken from the reaction and heated. After final centrifugation (5 min at 15,000× g), invertase activity was measured as Fru increase on HPAEC-IPAD. Enzyme activity was defined in nmol Fru produced from Suc min^−1^ g FW^−1^.

### 2.6. ROS Burst Measurements

Based on the protocol of Albert et al. [101], ROS burst was measured using a luminol-based assay as described by Janse van Rensburg et al. [56], with minor modifications. Mature leaves of untreated 4- to 5-week-old plants were used. A 3.5 mm cork-borer and plastic rack with 3.7 mm holes was used to punch leaf disks in the afternoon. Leaf disks were placed in a 96-well plate on 150 µL sterile ddH_2_O, covered with aluminum foil and incubated in the Conviron^®^ growth chamber under previously described conditions. After 16 to 18 h, the solution was replaced with 100 µL incubation solution, containing 20 µM luminol L-012 (Wako Chemicals, Richmond, VA, USA) and 1 µg mL^−1^ horseradish peroxidase (AppliChem, Darmstadt, Germany). Luminescence was measured using a GloMax^®^-Multi Detection System (Promega, Madison, WI, USA). Background luminescence was measured for 30 min after addition of incubation solution. Then, 100 µL assay solution was added, consisting of incubation solution and elicitor, and luminescence measured for 70 min at 1 min intervals and a 0.5 s integration time. As a positive control, 100 nM flg22 was used, while ddH_2_O was used as negative control. P95 chicory FOS, *H. smyrnensis* LOS and sulfated LOS were used at 5 g L^−1^ and 0.5 g L^−1^. Luminescence values were given as relative light emitting units (RLU) and background readings were subtracted from the elicitor-induced values. Total area under the curve was measured through integration methods.

### 2.7. PAL Assay

To measure PAL activity, we used a protocol modified from Liu et al. [102], measuring the production of cinnamic acid on HPLC. Extraction was performed on ice by adding 50 mg grinded sample to 300 µL extraction buffer containing 50 mM borate pH 8.5, 5 mM β-mercaptoethanol and 7% polyvinylpyrrolidone. Samples were centrifuged for 20 min at 20,000× *g* and 4 °C, after which supernatant was used to set up the reactions. A total of 35 mM phenylalanine was added to each sample and incubated at 30 °C for 1 h. Reactions (total volume 90 µL) were stopped by adding 10 µL HCl. Blank samples were taken from each reaction. Samples were diluted appropriately and analyzed on HPLC. An isocratic method was used with 50% MeOH and 50% ddH_2_O acidified to pH 2.7 using phosphoric acid. A Hypersil-keystone BDS Hypersil C18 column (150 mm × 4.6 mm) and UV detection at 260 nm were used. Protein concentration of extracts was measured as described for the chitinase assay. Enzyme activity was defined as the production of cinnamic acid in nmol min^−1^ mg^−1^ FW.

### 2.8. Myrosinase Assay

Fifty mg of grinded material was added to ice-cold extraction buffer, consisting of 50 mM MES pH 6.5. Samples were homogenized with a micropestle, followed by centrifugation for 20 min at 4 °C and 20,000× *g*. Two µL supernatant was added to the reaction mixture containing 2 mM sinigrin and 2.5 µM ascorbate. Incubations were run at 30 °C for 20 min, after which reactions were stopped by heating to 95 °C for 5 min. Blank control reactions were prepared by immediately heating the reaction mixture after adding the sample extract. Protein concentration of extracts was measured using the Bradford method [103]. A standard curve was prepared with bovine serum albumin (BSA). Myrosinase activity was analyzed by measuring Glc production on HPAEC-IPAD. Enzyme activity was expressed as nmol Glc min^−1^ g^−1^ FW.

### 2.9. Apoplastic Fluid Extractions

Following a protocol adapted from previous publications [104,105], apoplastic fluid (AF) extractions were performed on untreated, primed or infected leaves, depending on the experimental setup. Primed or infected leaves were rinsed before further manipulation to remove residual priming solution or infection buffer droplets. Leaves were completely submerged in ice-cold infiltration buffer consisting of 20 mM NaOAc pH 5.0, 400 µM rhamnose and 100 µM PMSF. Vacuum infiltration was performed in a vacuum flask by applying vacuum two times at 45 mbar for 2–5 min. Leaves were then rinsed with ddH_2_O to remove excess infiltration buffer and carefully blotted dry with paper towel. Dried leaves were rolled up into a piece of parafilm for rigidity during centrifugation and placed in a 5 mL syringe inside a 14 mL tube. Samples were centrifuged in a cooled swing bucket centrifuge for 10 min at 2000 rpm. The fluid collected in the 14 mL tube was transferred to clean 1.5 mL Eppendorf tubes and placed on ice.

To measure small soluble sugars, AF was boiled at 100 °C for 10 min and purified as described above using a Dowex^®^ column containing H^+^ and Ac^−^ resins. Samples were analyzed on HPAEC-IPAD. The apoplast dilution factor (ADF), describing how much the AF is diluted with the infiltration buffer, was measured by analyzing the rhamnose levels in the samples and comparing it to infiltration buffer control samples. To analyze fructan levels in AF samples, the Dowex^®^ flowthrough fraction was air-dried in a speedvac overnight and the pellet resuspended in a small volume of ddH_2_O, thus concentrating the sample for analysis on HPAEC-IPAD. The amount of LOS in AF was expressed as percentage relative to the highest observed peak areas.

### 2.10. Cytosol Contamination Assay

Full leaf extracts and AF samples were compared in this assay. The used enzymatic protocols were adapted from those described in Bergmeyer [106]. AF extractions were done as described in the previous section, except using a 50 mM Tris-HCl buffer pH 8.0 for the infiltration. This buffer was also used for extraction of grinded material of full leaves on ice with a micropestle. The samples were centrifuged (20 min at 20,000× *g* and 4 °C) and supernatant was used for further analysis. Samples were purified using sterile Sephadex G25 columns to isolate purified protein fractions. To measure malate dehydrogenase (MDH) activity, reactions were set up in Tris buffer pH 8.0 containing 350 µM β-NADH and 375 µM oxaloacetic acid. Reactions were performed at 21 °C in a 96-well plate and the decrease of OD_340_ was monitored every 10 s for 5 min. Phosphoglucoisomerase (PGI) assays were performed by incubating samples in Tris buffer pH 8.0 containing 1 mM β-NADP, 5 mM fructose-6-phosphate, 5 mM MgCl_2_ and 2.5 U mL^−1^ glucose 6-phosphate dehydrogenase. Increase in OD_340_ was measured for 30 min every 30 s at 21 °C in a 96-well plate. One U of activity was defined as the change in OD_340_ per min, taking into account the extinction coefficient of NADH. The percentage cytosol contamination was measured through comparison of enzyme activities between AF and full leaf extracts.

### 2.11. HPAEC-IPAD

Sugar samples were analyzed on a Dionex 5000 (Thermofisher Scientific, Waltham, Massachusetts, USA) HPAEC-IPAD. Ninety mM NaOH was used as mobile phase with a flow rate of 0.25 mL min^−1^, analyzing 6 µL sample on a CarboPac^TM^ PA100 column (Thermofisher Scientific, Waltham, MA, USA). Small soluble sugars were measured using an isocratic method of 90 mM NaOH for 10 min, while fructan samples were analyzed using a concentration gradient with NaOAc as described by Vergauwen et al. [99]. As an internal standard, rhamnose (Sigma-Aldrich, St. Louis, MO, USA) was added to all samples at a concentration of 20 µM. Glc, Fru and Suc standards of 10 µM (Acros Organics, Morris Plains, NJ, USA) were run alongside samples for peak identification and quantification.

### 2.12. Graphs and Statistics

Statistical analysis and graphs were performed in GraphPad Prism version 8.0.0 for Windows (www.graphpad.com; GraphPad Software, San Diego, CA, USA). A ROUT method was used to identify statistical outliers. Normality of the data was tested using Shapiro–Wilk and Anderson–Darling tests. Treatment comparison was analyzed with one-way ANOVA, followed by a Holm–Šídák multiple comparison test, or with a Kruskal–Wallis non-parametric test, followed by a Dunn’s multiple comparison test. A two-way ANOVA was used for assays with different time points of analysis with Tukey’s multiple comparisons test. Categorized data from the disease assay were analyzed using a two-tailed Mann–Whitney *u*-test.

## 3. Results

### 3.1. Optimizing Disease Assays in Rocket

**Figure 1:**
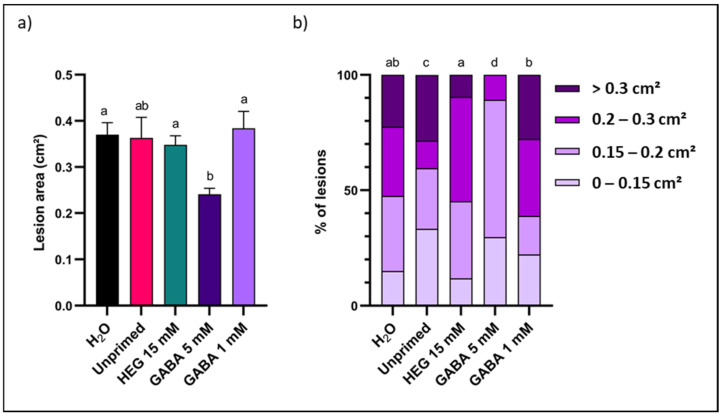
Validation of the priming-infection assay in the rocket-*Botrytis cinerea* pathosystem. After 72 h of priming, leaves were detached, rinsed and inoculated with *B. cinerea* spores. Infection progressed for 72 h before disease scoring. (**a**) Average lesion area of negative, osmotic (hexaethylene glycol, HEG) and positive control (γ-aminobutyric acid, GABA). Bars represent the mean ± SEM. (**b**) Percentage of lesions belonging to each of four different classes based on lesion size. A minimum of 30 biological replicates were used per treatment and the experiment was repeated three times with consistent results. Letters indicate significant differences between treatments (*p* < 0.05).

To the best of our knowledge, there are no reports on carbohydrate-mediated leaf priming studies on rocket. Prior to initiating fructan priming on rocket leaves, the priming and infection setup was verified using different control treatments. Since all fructan solutions were dissolved in ddH_2_O with 0.001% Tween-20, this was used as negative control. However, from preliminary data we learned that rocket can sometimes experience the water control as a stress situation, causing a hypo-osmotic shock, probably depending on the exact osmotic status of the apoplastic continuum at the moment of the priming treatments. To grasp the overall picture, the water control was compared to a HEG osmotic control at 15 mM, resembling the highest estimated molarity of the low DP fructan solutions at 5 g L^−1^. An unprimed control was also included, where leaves were not sprayed with priming solution, to verify the effects of water spraying as such. In most cases, no clear differences in disease resistance were observed between the three controls, after measuring *B. cinerea* lesion areas 72 h after spore inoculation (Figure 1a). Lesions were also classified into four classes based on lesion size (Figure 1b). The distribution was significantly different for unprimed leaves compared to negative and osmotic controls (Figure 1b). However, as demonstrated by the average lesion area data (Figure 1a), this difference marginally influences overall disease resistance.

GABA was considered to be a positive control treatment, showing promising effects in *A. thaliana* [53]. In rocket, GABA priming significantly improved pathogen resistance at 5 mM, while no effects were observed at 1 mM (Figure 1a). Classified lesion size data indicated the same differences (Figure 1b). After priming with 5 mM GABA, no lesions were observed with an area above 0.3 cm^2^, showing a distribution that was significantly different compared to all other treatments (Figure 1b).

### 3.2. Fructan Priming Solutions: Partial Degradations, Purificiation and Sulfated LOS Production

**Figure 2:**
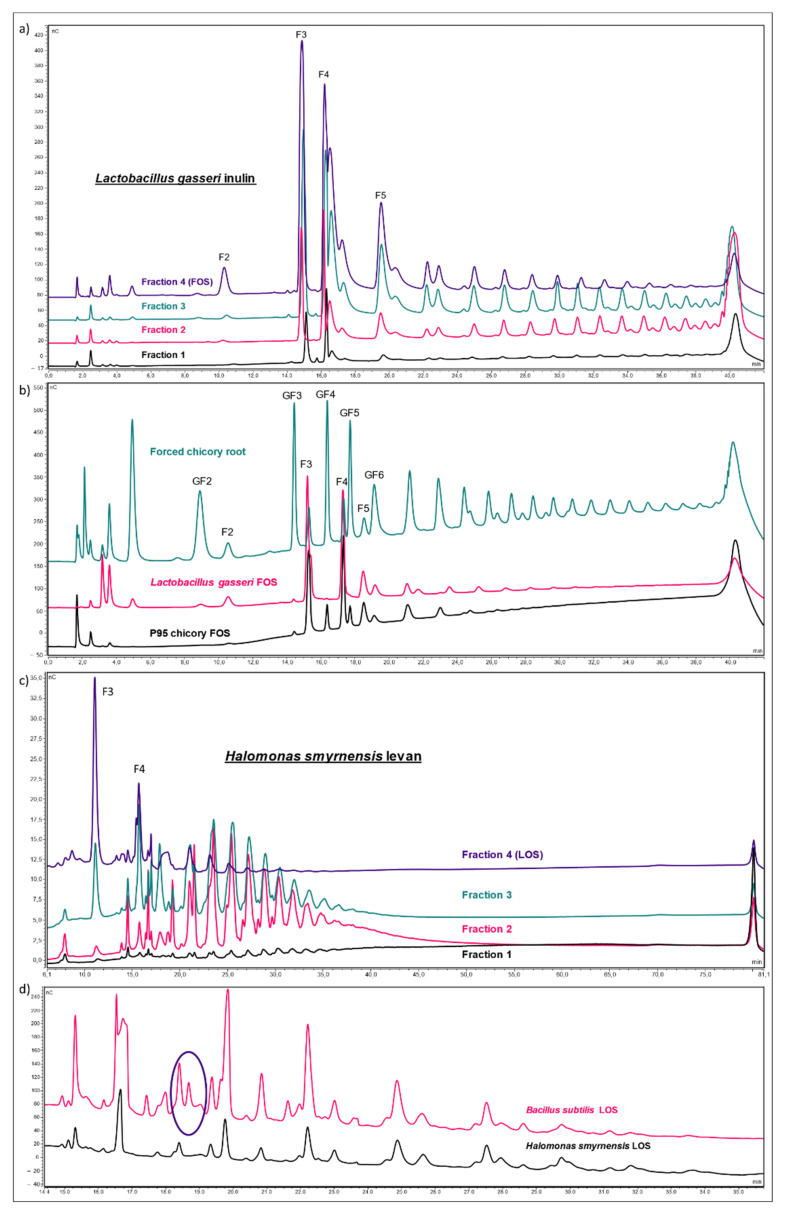
Different types of fructans used as priming agent and the production of fructan hydrolysis products. (**a**) *Lactobacillus gasseri* was hydrolyzed by *Aspergillus niger* endo-inulinase. Products were separated by degree of polymerization (DP) using acetone precipitation. (**b**) Fructooligosaccharide (FOS) profile of *L. gasseri* was compared to P95 chicory FOS and peaks identified with a forced chicory root reference sample. (**c**) A *Bacteroides thetatiotaomicron* endo-levanase was used to hydrolyze *Halomonas smyrnensis* levan into different DP fractions, fraction 4 resembling levan oligosaccharides (LOS). (**d**) Comparison between *H. smyrnensis* and *Bacillus subtilis* LOS fraction indicates a different degree of branching, exemplified in the purple circle. Samples were analyzed on HPAEC-IPAD. GFn peaks indicate inulin or levan of DP n + 1. Fn peaks indicate fructose-only type of fructans of DP n. X-axis indicates retention time (min), while y-axis shows the amperometric signal (nC).

Different inulin-type fructan solutions were prepared for priming. Commercially available chicory inulin and chicory fructooligosaccharides (FOS) were compared to microbial counterparts from *L. gasseri*. Commercial chicory FOS (termed P95) was obtained through partial hydrolysis of chicory inulin with a fungal endo-inulinase. For comparison, we produced a similar FOS fraction from the high DP *L. gasseri* inulin, using an endo-inulinase from *A. niger*. Reaction products were separated into four different fractions with different average DP (Figure 2a). Fraction 4 has the lowest average DP and represents the FOS fraction, which is similar to P95 FOS and consists mainly of inulotriose and inulotetraose, both Fru-only fructans of the Fn type. However, chicory FOS contains relatively higher levels of GFn fructans, such as GF4 and GF5 (Figure 2b).

In a similar way, levan of plant and microbial origin were compared. For the microbial levan, the high DP fraction was further compared with LOS fractions. Using an endo-levanase enzyme from *B. thetaiotaomicron*, both *H. smyrnensis* and *B. subtilis* levan were partially degraded, producing again four fractions of different average DP. The endo-levanase was successfully expressed in *E. coli* (Figure S1a) and the observed catalytic activities confirmed the affinity for levan, but not for inulin, in line with Mardo et al. [95]. Very low Suc-hydrolyzing activity was also observed (Figure S1b). Figure 2c shows the four fractions of *H. smyrnensis* hydrolysis, with fraction 4 resembling the LOS fraction used for priming experiments. A comparison was made with LOS from *B. subtilis*, since the latter is more branched as compared to the more linear *H. smyrnensis* levan [79]. This was confirmed here by the more complex profiles of the *B. subtilis* LOS (Figure 2d). Graminan from *T. aestivum* and agavin from *A. tequilana* were also considered in priming experiments (Figure S2). Fractions containing considerable contamination with small soluble sugars (Glc, Fru, Suc) were further purified before use as priming agent. Chromatograms of purified fructans can be found in Figure S3.

**Figure 3:**
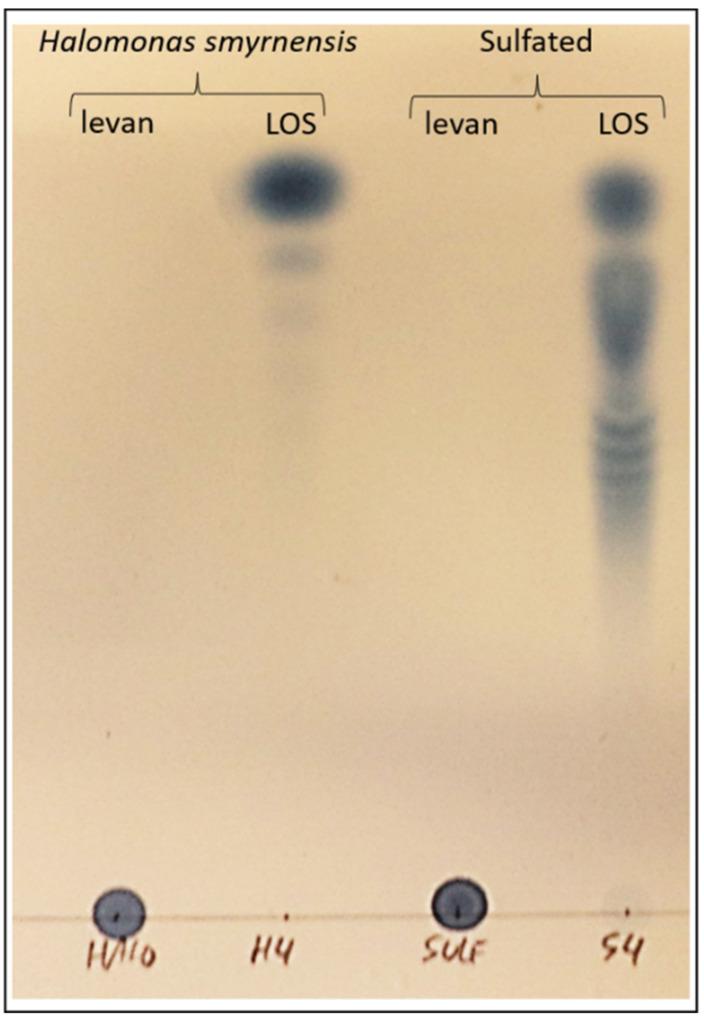
Sulfated levan can be hydrolyzed by a microbial endo-levanase. Similar to *Halomonas smyrnensis* levan, the sulfated levan variant was hydrolyzed by *Bacteroides thetaiotaomicron* endo-levanase. LOS fractions were purified and analyzed with TLC.

Finally, a sulfated *H. smyrnensis* levan was also considered, and the sulfated LOS obtained after hydrolysis was compared to its non-sulfated counterpart. To the best of our knowledge, the catalytic activity of a levanase has never been tested on sulfated levan. Enzyme addition revealed a significant increase in sulfated LOS. Sulfated LOS in the original levan sample was close to zero and accounted for 3.91% of the total weight without addition of an endo-levanase in the incubation. When the enzyme was added, almost half of the total weight after incubation consisted of sulfated LOS (43.02%). Since further analysis of this LOS fraction was not possible on HPAEC-IPAD due to the negative nature of the sulfate groups, TLC methods were used. A comparison with *H. smyrnensis* levan and LOS further confirmed the production of sulfated LOS (Figure 3).

### 3.3. LOS Priming Increases Rocket Resistance to B. cinerea Infection

**Figure 4:**
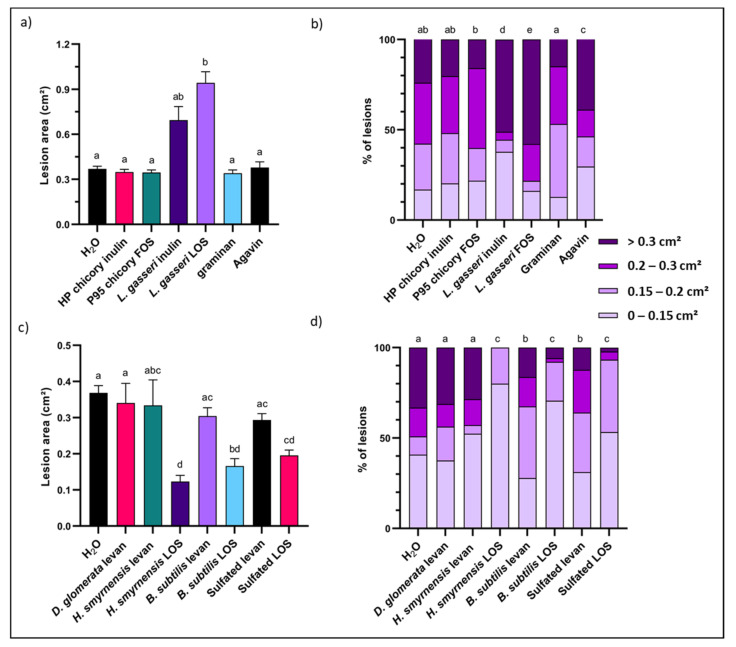
Priming efficacy of an array of fructans in the rocket-*Botrytis cinerea* pathosystem. After 72 h of priming, leaves were detached, rinsed and inoculated with *B. cinerea* spores. Infection progressed for 72 h before disease scoring. (**a**) Average lesion area for different inulin-type fructans (chicory or *Lactobacillus gasseri*), as well as *Triticum aestivum* graminan and *Agave tequilana* agavin. (**b**) Percentage of lesions belonging to each of four different classes based on lesion size for inulin and branched fructans. (**c**) Average lesion area of levan priming agents (*Dactylis glomerata*, *Halomonas smyrnensis* and *Bacillus subtilis*). For *H. smyrnensis*, a sulfated levan was also tested. Bars represent the mean ± SEM. (**d**) Percentage of lesions belonging to each of the four classes with different lesion size for levan and LOS priming. All fructans were sprayed at 5 g L^−1^, with at least 30 biological replicates per treatment. The experiments were repeated three times with consistent results. Letters indicate significant differences between treatments (*p* < 0.05).

Rocket leaves were primed with the different fructan solutions at 5 g L^−1^ and compared to the control. None of the plant-derived fructans had a significant impact on disease resistance, independent of linkage type or branching (Figure 4a,c). Curiously, microbial inulin, and especially *L. gasseri* FOS increased susceptibility of the leaves to infection. Classified lesion size data showed the same trends with significant changes in distribution for microbial inulin and LOS, characterized mainly by a strong increase in lesions belonging to the largest size class (Figure 4b). Graminan and agavin were significantly different from the control treatment in terms of size distribution, although overall disease resistance remained unchanged (Figure 1a).

*H. smyrnensis*, *B. subtilis* and sulfated levan were all similar to the control, as was the case for *D. glomerata* levan (Figure 4c). All the microbial LOS fractions, on the other hand, significantly improved rocket disease resistance (Figure 4c). The class distribution (Figure 4d) showed the same trends, although here *B. subtilis* and sulfated levan were significantly different from *H. smyrnensis* and *D. glomerata* levan, as well as from the control. The difference was found mainly in the abundance of lesions falling into size category 2 and 3, although the effect on average lesion size was minimal. No significant differences were observed between LOS treatments and all three were characterized by very low abundance of lesions falling into the largest size category. Strikingly, for *H. smyrnensis* LOS, large lesions were completely absent (Figure 4d).

### 3.4. Effect of Priming Dosage, Fructan DP and Priming Duration on LOS Priming

**Figure 5:**
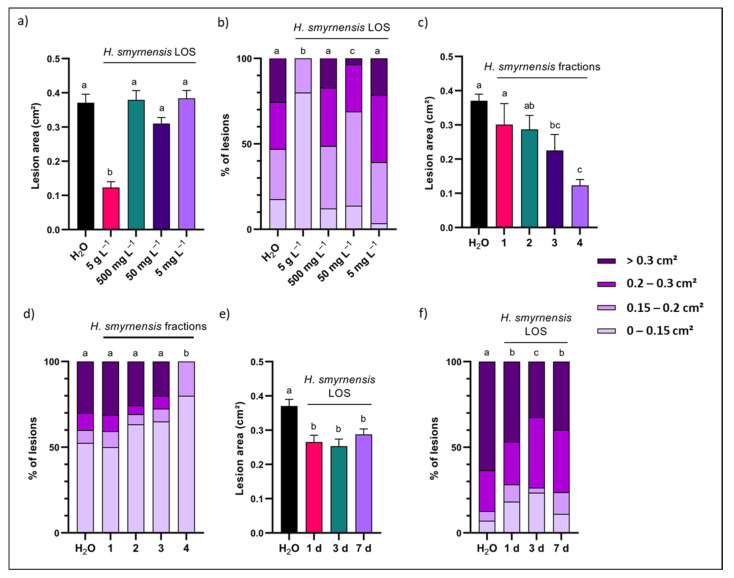
Influence of concentration, degree of polymerization (DP) and priming duration of fructans in the rocket-*Botrytis cinerea* pathosystem. After 72 h of priming (unless denoted otherwise), leaves were detached, rinsed and inoculated with *B. cinerea* spores. Infection progressed for 72 h before disease scoring. (**a**) Average lesion area of leaves sprayed with different concentrations of *Halomonas smyrnensis* levan oligosaccharides (LOS). (**b**) Percentage of lesions belonging to four different classes based on lesion size after priming with different concentrations of LOS. (**c**) Average lesion area for different levan fractions produced from *H. smyrnensis* levan degradation. Fraction 4 resembles the LOS fraction. (**d**) Percentage of lesions belonging to each lesion size class for different *H. smyrnensis* fractions. (**e**) Average lesion areas of rocket leaves infected at different time points after LOS priming. Bars represent the mean ± SEM. (**f**) Percentage of lesions belonging to four different classes based on lesion size, with *B. cinerea* spore inoculation at different time points after priming. Fructans were sprayed at a concentration of 5 g L^−1^ unless specified. At least 30 biological replicates were used for each treatment and the experiments repeated three times with consistent results. Letters indicate significant differences between treatments (*p* < 0.05).

To study the optimal concentration for priming, *H. smyrnensis* LOS was used, since it was the best-performing fraction (Figure 4c,d). Lower concentrations were tested in the range of 5–500 mg L^−1^ and compared to 5 g L^−1^. None of the lower concentrations improved rocket resistance (Figure 5a,b). Thus, 5 g L^−1^ was used in follow-up experiments.

Interestingly, when comparing all four DP fractions produced during enzymatic breakdown of *H. smyrnensis* levan, a clear effect on priming efficacy was observed. Fractions 3 and 4 were significantly more effective compared to the control condition and to fraction 1 (highest DP; Figure 5c). Fraction 2 and 3 differed only marginally, while the LOS fraction 4 performed significantly better than all treatments (except fraction 3). Lesion size distributions showed a similar trend, although less pronounced. Here, fraction 4 (LOS) improved rocket disease resistance significantly compared to all other treatments (Figure 5d). These data show a clear trend that lower DP levan-type fructans have higher priming potential.

Next, a priming duration of 72 h was compared to shorter and longer time windows for the *H. smyrnensis* LOS treatment. Our results indicate a significant effect of priming already after 1 d, and lasting at least for 7 d (Figure 5e). No difference between time points is observed for average lesion area, although lesion distribution data indicated that the priming effect was significantly higher after 3 d (Figure 5f). These data give a first indication of the lasting effect of fructan priming for rocket against subsequent stress exposure, indicating a potential optimal response around 3 d after the priming treatment.

### 3.5. H. smyrnensis LOS-Primed Leaves Associate with Higher Sugar Contents

The sweet immunity concept describes a significant role for small soluble sugars in plant immune responses [78]. Therefore, sugar dynamics were thoroughly followed in rocket leaves in response to priming and subsequent *B. cinerea* infection, alongside an infection control (IC), representing leaves incubated with infiltration buffer without spores. Besides measuring Glc, Fru and Suc, starch content was also measured through complete hydrolysis and measurement of the produced Glc. All samples were analyzed on HPAEC-IPAD. Total hexose, total sugar and Suc/hexose ratios were calculated as well.

**Figure 6:**
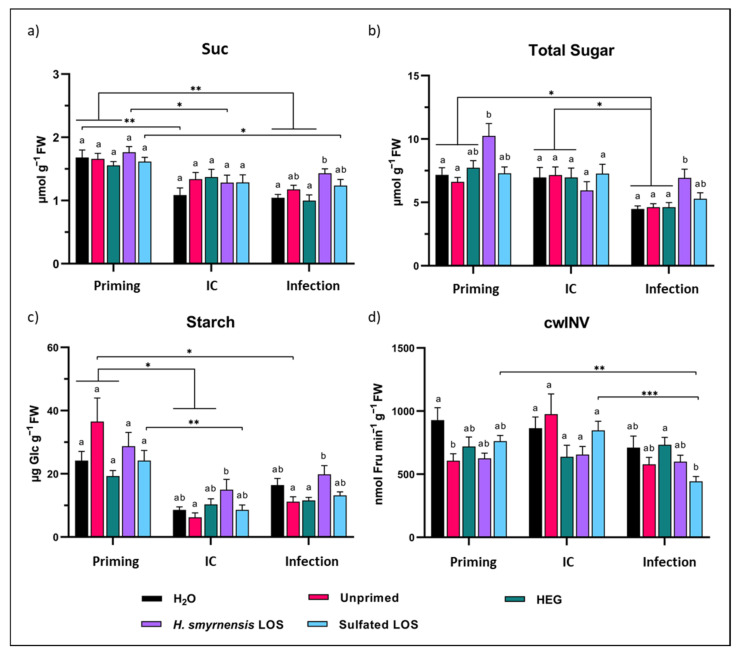
Effect of fructan priming on small soluble sugars, starch and cell-wall invertase (cwINV) in the rocket-*Botrytis cinerea* pathosystem. Glucose, fructose and (**a**) sucrose (Suc) levels were measured on HPAEC-IPAD. (**b**) Total sugar was calculated based on these results. (**c**) Starch content was measured by following Glc production due to starch degradation. (**d**) cwINV activity was assayed as the increase in Fru from Suc hydrolysis. Samples were taken after priming or after inoculation with infection buffer (infection control, IC) or infection buffer with fungal spores. Bars represent the mean ± SEM. A total of 6 biological replicates were used per treatment and the experiment was repeated three times with consistent results. Letters indicate significant differences between treatments within the same time point (*p* < 0.05). Asterisks indicate significant differences of the same treatment between time points (* *p* < 0.05; ** *p* < 0.01; *** *p* < 0.001).

Overall, Glc levels were much higher than Fru and Suc for all conditions (Figure S4a and Figure 6a). The different priming treatments had no significant effect on Glc levels, although the concentration was visibly higher for *H. smyrnensis* LOS-treated leaves after priming (Figure S4a). Glc levels decreased after infection, compared to IC conditions. This effect was not apparent for Fru, although a significant decrease in concentration was observed between primed and infected leaves for unprimed and HEG-primed leaves (Figure S4b). Sulfated LOS-primed leaves had significantly lower Fru compared to other treatments, except for H_2_O and unprimed control conditions (Figure S4b). After priming, Suc levels remained unchanged between the treatments (Figure 6a). After infection, Suc levels in LOS-treated plants were significantly higher compared to H_2_O- and HEG-primed plants. Suc concentrations decreased significantly for H_2_O, unprimed and HEG-primed conditions between priming and infection (Figure 6a). In the case of H_2_O, this decrease was also observed for IC conditions (Figure 6a).

Total small soluble sugar levels showed marked differences in LOS-treated samples after priming and infection, but not in the IC (Figure 6b). Moreover, while sugars declined significantly after infection for the IC, no significant decrease was measured in LOS-primed leaves. Total hexose levels showed very similar trends, in line with total sugar levels (Figure S4c). Differences in Suc/hexose levels depending on priming treatment were minimal (Figure S4d).

Starch content in the leaves decreased markedly after leaf detachment (comparing IC and infection; Figure 6c). A significant decrease was found for H_2_O, unprimed and HEG-treated leaves. After LOS priming, starch content in IC and infected leaves was higher compared to other treatments, although a level of significance was reached compared to unprimed leaves and HEG priming, but not to H_2_O treatment. These data indicate a decrease in total sugar and starch contents for the three control conditions after leaf detachment and/or infection. For fructan-primed leaves, this decrease was less apparent, and especially in the case of LOS treatment, significantly higher levels of small soluble sugar and starch were maintained (Figure 6b,c).

### 3.6. Fructan Priming Has No Clear Effect on Plant Invertase Activities

Acid invertases, and especially cwINVs, are important in regulating Suc/hexose ratios during pathogen infection. Both vINV and cwINV activities were measured on samples taken from rocket leaves using the same experimental setup as described above. However, no clear differences were observed in terms of vINV activity (Figure S4e), having significantly lower activity than cwINV. After priming, cwINV activity was higher after H_2_O priming compared to unprimed conditions (Figure 6d). cwINV activity was significantly different for HEG- and sulfated LOS-primed samples after infection. Finally, cwINV activity decreased significantly after infection (compared to priming and IC conditions) for sulfated LOS-primed plants (Figure 6d).

### 3.7. Priming with (Sulfated) LOS Affects Plant Immune Signaling

Although a priming stimulus itself has relatively low fitness costs and low induction of gene expression, it will lead to a stronger immune response after subsequent stress exposure. Therefore, PAL was measured as a potential central immune player in the rocket-*B. cinerea* interaction after priming as well as subsequent infection, testing IC alongside as a control. PAL is a key enzyme in the production of many secondary metabolites, often involved in biotic stress resistance. Moreover, it is involved in the production of SA. Specifically, in Brassicaceae species, myrosinases degrade glucosinolates into Glc and toxic by-products in case of cellular damage.

**Figure 7:**
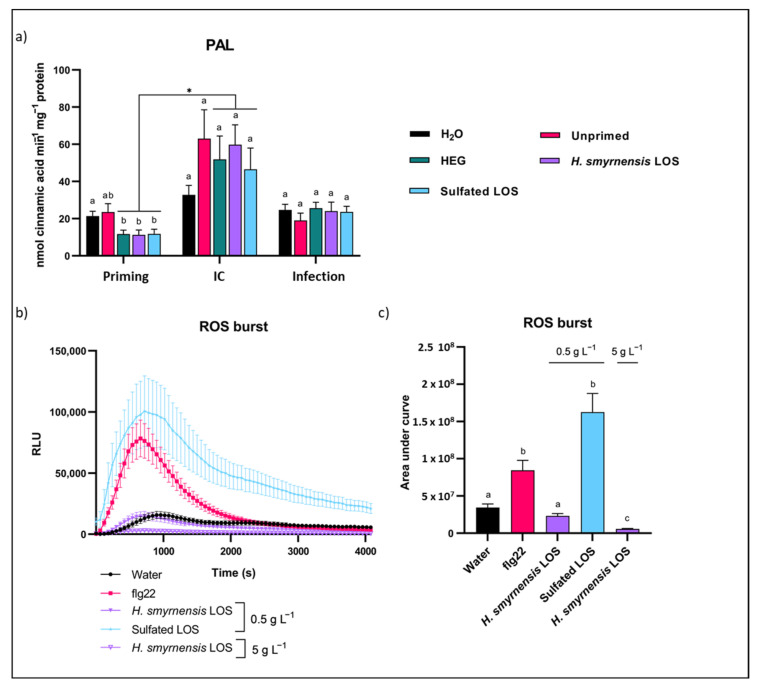
The effect of fructan priming on immunity-related parameters in the rocket-*Botrytis cinerea* pathosystem. (**a**) Activity of phenylalanine ammonia-lyase (PAL) was measured through production of cinnamic acid on HPLC. Samples were taken after priming or after subsequent infection. Infection control (IC) buffer, without spores, was also used for inoculation. (**b**) The direct effect of fructans as elicitor on the ROS burst was tested, using flg22 as positive control. Unprimed plants were used. Data are expressed as relative light emitting units (RLU). (**c**) Total area under the ROS burst curve was calculated to compare treatments. Bars represent the mean ± SEM. At least 6 (**a**) or 8 (**b**,**c**) biological replicates were used, and the experiments were repeated 3 times with consistent results. Letters indicate significant differences between treatments (*p* < 0.05). Asterisks indicate significant differences between time points for the same priming agent (* *p* < 0.05).

No significant differences were observed for the different priming treatments in terms of Glc production from myrosinase activity (Figure S5). At 24 h after priming, activity was visibly higher for HEG and LOS, which could indicate an osmotic effect, rather than a fructan-specific effect. In the control condition, activity increased significantly after detachment. After infection, Myrosinase activity was significantly higher for LOS-primed leaves compared to HEG treatment. Priming significantly influenced PAL activity, with reduced activity in HEG-, LOS- and sulfated LOS-primed leaves, as compared to the negative control (Figure 7a). Subsequently, a significant induction of activity was found for these treatments after leaf detachment. After infection, PAL activities visibly dropped compared to IC (*p*-value for *H. smyrnensis* LOS between IC and infection = 0.0596). This near-significant status can be explained by the very high variation in the IC samples. High variation is typically observed in systemic wounding processes such as leaf cutting [107,108].

Next, the direct effect of these fructans on the production of an apoplastic ROS burst was tested on unprimed leaves, to discern between a priming effect or a direct immune system elicitation. Different concentrations of LOS were considered, although technical issues arose when using 5 g L^−1^ sulfated LOS. H_2_O and flg22 were used as negative and positive control, respectively. Flg22 treated controls showed a clear ROS burst induction, as expected, compared to H_2_O. *H. smyrnensis* LOS showed no immediate induction of a burst (Figure 7b). Sulfated LOS, on the contrary, showed a burst similar to flg22 in terms of height, but with a slower return to basal levels. Burst area data confirmed these differences, showing a significant difference between H_2_O and flg22 (Figure 7c). Sulfated LOS was significantly higher than all treatments except flg22. LOS priming showed a significantly lower burst compared to flg22 for both concentrations tested.

### 3.8. Apoplastic Sugars Decrease after B. cinerea Infection

**Figure 8:**
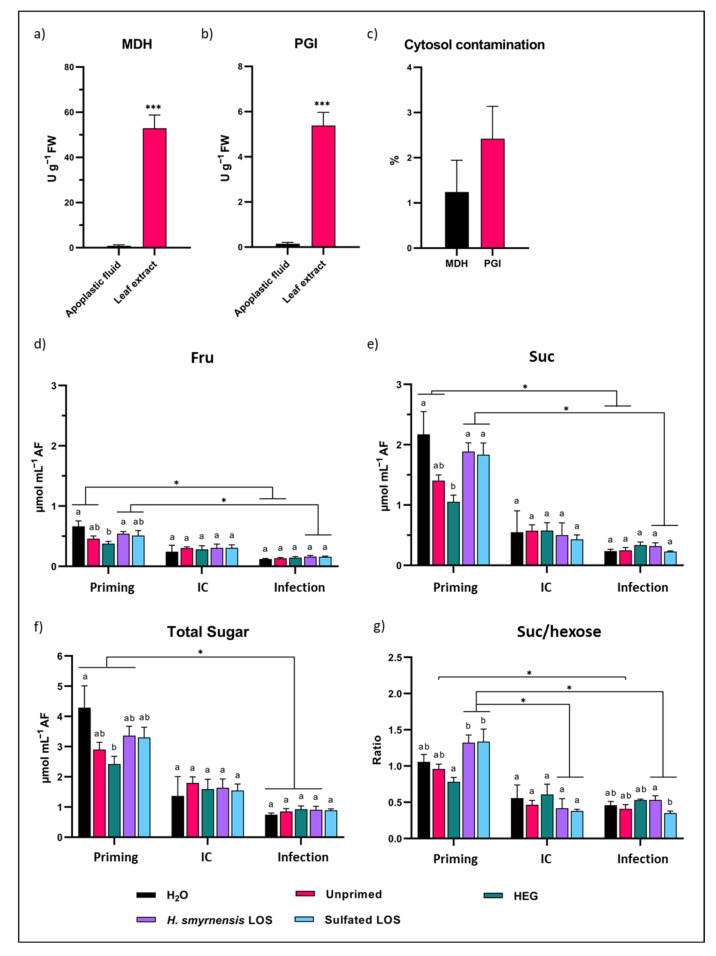
Measuring apoplastic sugars after fructan priming in the rocket-*Botrytis cinerea* pathosystem with low cytosol contamination rates. Apoplastic fluid (AF) was extracted through vacuum infiltration-centrifugation techniques. Cytosol contamination was tested by comparing activity of (**a**) malate dehydrogenase (MDH) and (**b**) phosphoglucoisomerase (PGI) activity of AF and full leaf extracts. (**c**) Cytosol contamination levels were calculated as the percentage activity in AF compared to full leaf. Levels of (**d**) fructose (Fru) and (**e**) sucrose (Suc) were measured from AF samples on HPAEC-IPAD and (**f**) total sugar and (**g**) Suc/hexose ratio were calculated. Samples were taken from primed or infected leaves. Infection control (IC) samples were also included. Bars represent the mean ± SEM. At least 6 biological replicates were used per treatment. The cytosol contamination assay was repeated 3 times with consistent results. Letters indicate significant differences between treatments within the same time point (*p* < 0.05). Asterisks indicate significant differences of the same treatment between time points or between extraction methods (* *p* < 0.05; *** *p* < 0.001).

During plant–pathogen interaction, apoplastic sugar dynamics are important in determining the outcome of infection. Apoplastic sugars can serve as the main carbon source for a pathogen, especially at the early stages of infection, even for necrotrophs, which are believed to have an initial but short biotrophic phase as well [109]. An apoplast extraction method was optimized for rocket based on the vacuum infiltration technique. To control for cytosolic contamination in AF extracts, enzyme assays were carried out for two intracellular enzymes with a central role in primary metabolism. Activities were compared to those in full leaf extracts. In the case of both MDH and PGI, activities were significantly lower in AF samples (Figure 8a,b). When expressed as percentage activity in AF compared to full leaf, average values were below 3%, indicating limited cytosol contamination (Figure 8c).

Small soluble sugars were further purified from AF samples and analyzed on HPAEC-IPAD. No differences were observed between priming treatments for Glc (Figure S6a). For both Fru and Suc, however, apoplastic levels were significantly higher for LOS and sulfated LOS priming compared to HEG (Figure 8d,e). Intriguingly, apoplastic concentrations for both sugars were also significantly different when comparing H_2_O- and HEG-primed leaves. The same effects were also observed for total sugars (Figure 8f). When comparing primed and infected leaf samples, a significant decrease in apoplastic Glc and total hexose was observed for all priming treatments except sulfated LOS (Figure S6a,b). For Fru, Suc and total sugars, this reduction was significant for all treatments used, except for HEG. Apoplastic Suc/hexose ratios were higher after *H. smyrnensis* and sulfated LOS priming, especially compared to HEG-treated samples (Figure 8g). For both these treatments, the ratio decreased significantly after leaf detachment (IC and infected leaves).

### 3.9. Apoplast Dynamics after LOS and FOS Priming

**Figure 9:**
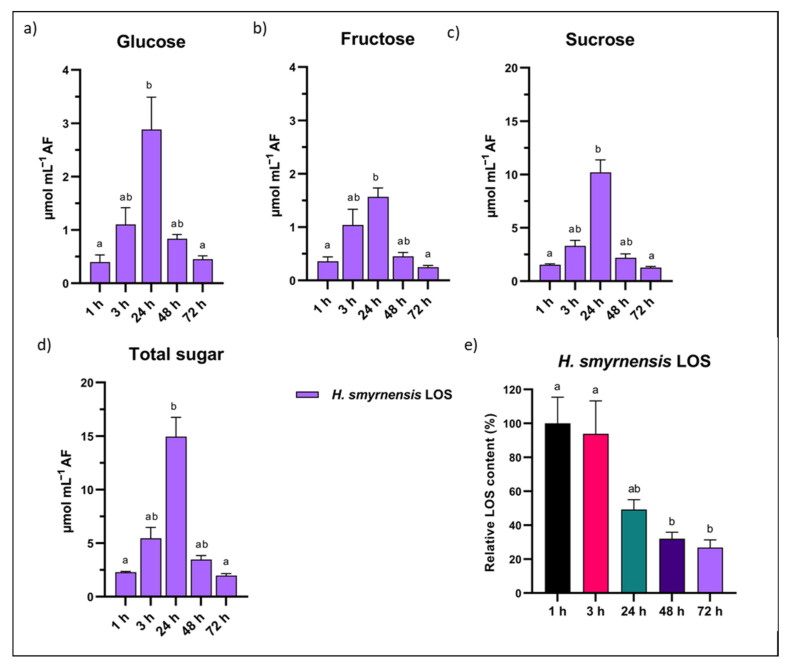
Apoplastic dynamics after LOS priming in rocket leaves. Apoplastic fluid (AF) was extracted from rocket leaves at different time points after LOS priming. Apoplastic (**a**) Glc (**b**) Fru and (**c**) Suc were measured on HPAEC-IPAD and (**d**) total sugar levels were calculated. (**e**) LOS profiles were analyzed on HPAEC-IPAD after LOS priming. Bars represent the mean ± SEM. A minimum of 6 biological replicates was used per treatment. The experiment was repeated 2 times with consistent results. Letters indicate significant differences between time points (*p* < 0.05).

The effect of LOS priming on apoplastic small soluble sugars was studied in more detail by taking additional time points after the priming treatment. Suc was again the most abundant small soluble sugar. For Glc, Fru and Suc, a peak in apoplastic concentration was observed 24 h after LOS priming (Figure 9a–c). As a result, total sugar levels showed the same trend (Figure 9d). The increase was significant when comparing 1 h and 24 h time points. After 24 h, apoplastic levels decreased, returning to normal values at 72 h, similar to the 1 h time point. Concomitantly, this decline is significant between 24 h and 72 h. This indicates a peak in apoplastic small soluble sugars after 24 h, followed by a significant decline in LOS-treated samples.

When leaves are sprayed with LOS, a percentage of the exogenous fructans is expected to reach the apoplast, where they could potentially function as signaling compounds sensed by so far uncharacterized receptors. To confirm the presence of LOS in the apoplast after priming, fructan levels were measured from AF on HPAEC-IPAD. Leaves were carefully rinsed before AF extractions to prevent contamination from LOS residue on the outside due to spraying. Our results clearly indicate the presence of LOS in AF already 1 h after priming (Figure 9e). Levels remained constant in the early time points, but started to decrease later. LOS levels significantly declined after 48 h and 72 h, probably through the action of apoplastic 6-FEHs.

**Figure 10:**
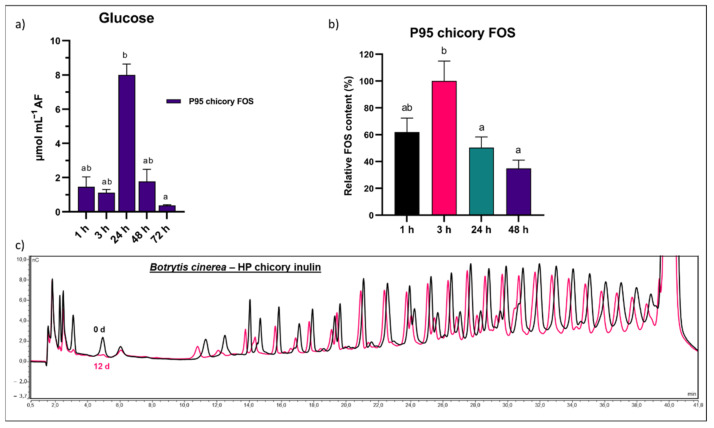
Apoplastic FOS dynamics after priming in rocket leaves. Apoplastic fluid (AF) was extracted from rocket leaves at different time points after priming. (**a**) Apoplastic Glc levels were measured at different time points after FOS priming. (**b**) FOS levels in AF at different time points after FOS priming. Bars represent the mean ± SEM. A minimum of 6 biological replicates was used per treatment. The experiment was repeated 2 times with consistent results. Letters indicate significant differences between time points (*p* < 0.05). (**c**) Incubation of *B. cinerea* in minimal medium supplemented with HP chicory inulin. Inulin degradation and consumption by the fungus were analyzed on HPAEC-IPAD after 12 d. X-axis shows retention time (min), y-axis indicates amperometric signal (nC).

The interesting effects of LOS priming on apoplastic fluid levels led us to test apoplastic sugar dynamics in the case of P95 FOS priming, a fructan substrate with similar DP but different linkage type. Interestingly, we observed a strong increase in apoplastic Glc after 24 h (Figure 10a), likely at least partially originating from the degradation of FOS of the GFn type (Figure 10b). The increased apoplastic sugar levels in combination with the presence of left-over apoplastic FOS at the infection time (24 h) may be beneficial for the pathogen. Our incubations of *B. cinerea* B05.10 with HP chicory inulin confirmed the consumption of mainly low DP FOS, although peaks of higher DP inulin were also reduced after a very long time interval (12 d) on minimal medium (Figure 10c).

## 4. Discussion

The use of biologicals can reduce pesticide usage in light of integrated pest management strategies, functioning against biotic stressors or directly stimulating immune responses of the plant [4,5]. There is an increasing need for such alternatives due to an increasing demand for food production and the negative effects of current pesticide usage [3]. In this regard, several benefits exist in using natural products as priming agents, since they are readily available, cheap, and less harmful to both human health and the environment [3,8,54]. In this study, fructans were considered to be priming agents, since earlier studies confirmed that fructans can increase resistance of several plants to pathogen infection [8,56,76,78,110]. Priming efficacy was tested for a broad array of different fructan types on leaves of rocket, a close relative to *A. thaliana* and an economically important healthy vegetable. For the most promising fructan types, effects on carbohydrate metabolism, immune system and (apoplast) sugar dynamics were investigated.

Priming with chicory inulin fractions or branched fructans had no effect, while a negative effect was observed for microbial inulin (Figure 4a,b). This fits with data obtained for chicory inulin in the *Venturia*/apple pathosystem [78] but differs from *L. sativa* and *A. thaliana/Botrytis* pathosystems [8,56], where inulin effectively reduced lesion size of *B. cinerea*. Moreover, several studies with inulin from *Arctium lappa* indicate a positive effect of plant inulin priming on host resistance [76,111]. In fructan-accumulating plants, such as *L. sativa*, inulin may be sensed by the host as a DAMP, released to the extracellular environment during cellular rupture [81]. Why inulin boosts defense responses in *A. thaliana* but not in rocket is unclear, since neither species produces endogenous inulin, making DAMP-type recognition unlikely. If fructan priming would influence the microbial community of the phyllosphere, rather than being recognized directly by the plant cell, the observed differences may involve the presence of a different microbial composition. It is predicted that some epi- and endophytic micro-organisms can use inulin, equivalent to the case of the gut microbiome [112,113], and as such, inulin priming may induce a change in microbial composition. Nevertheless, much more research is necessary in this area to understand if and how inulin priming can influence phyllosphere microbial communities.

*L. gasseri* inulin visibly reduced disease resistance after priming, while *L. gasseri* FOS showed an even stronger negative effect (Figure 4a,b). The higher DP microbial inulin is expected to be less mobile in the apoplast as compared to FOS. Thus, FOS that migrates deeper into the apoplast cannot be rinsed from the leaves after priming while the higher DP inulin remaining on the leaf surface can be removed by washing. Some apoplastic FOS and other metabolizable substrates such as Glc are still present at the time of *B. cinerea* infection (24 h), stimulating pathogen virulence and growth (Figure 10a,b). Indeed, inulinase activity was recently discovered for *B. cinerea* [114]. Typically, fungal inulinases show a higher affinity towards FOS as compared to inulin, and this is also the case for *B. cinerea* (Figure 10c).

When considering all available manuscripts on fructan prebiotic or immune-stimulating effects on animals, the branched graminans and agavins received limited attention [115]. In plant research, priming data for branched fructans are scarce. Therefore, we tested *T. aestivum* graminan and *A. tequilana* agavin in our rocket-*B. cinerea* pathosystem. Yet, no effects were observed with these fructan priming agents, in contrast to branched plant or fungal β-glucans that function as recognized MAMPs or DAMPs. Higher immunostimulatory activity was observed for branched oligosaccharides compared to linear ones [14,116,117]. Recognition of mixed-type fructans as DAMP will be limited to species harboring endogenous mixed fructans (such as wheat), and MAMP recognition is unlikely, given the absence of clear microbial mixed-type fructan production. It will, however, be interesting to obtain graminan with a similar DP as the bio-active β-glucans and make a full comparison in rocket and other plant species.

The most interesting results were observed for the LOS priming agents, while higher DP levans from *H. smyrnensis* had no clear influence on disease resistance (Figure 4c,d). Although this levan-type fructan moderately improved resistance of *A. thaliana* to infection, it was much less effective than LOS, while plant-derived medium DP levans also proved ineffective [56]. In the case of rocket there was a small reduction in disease symptoms after priming with *B. subtilis* or sulfated levan. This very high DP branched and sulfated levan may have a reduced solubility during leaf rinsing, and the remaining levan residue may shield the leaf surface during *B. cinerea* infection. On the contrary, plant-derived medium DP levans caused positive effects on resistance in the *Venturia*/apple pathosystem [78]. This may indicate different responses between host species, similar to the observed differences for inulin priming. However, the lifestyle of the pathogen may also be a strong influencing factor, since *Venturia* is a hemibiotroph.

More importantly, in line with the observations of Janse van Rensburg et al. [56] on *A. thaliana*, LOS priming was identified as the most effective fructan priming agent in rocket as well. All three types of LOS considered here significantly reduced lesion size after infection, accompanied with the (near) absence of lesions with an area larger than 0.2 cm^2^. Compared to levan (especially of microbial origin), LOS are more mobile priming compounds. Our results confirm earlier predictions on the potential signaling role of LOS in plants [81]. Since levan is produced as an exopolysaccharide by many bacteria, it may be recognized by the plant as MAMP. The higher efficacy of LOS priming indicates partial hydrolysis of microbial levan during plant-microbe interaction. Although microbial endo-levanases are likely involved, further trimming of the produced levan-nose type fructans (Fru-only fructans without a terminal Glc) may involve plant 6-FEH enzymes. Further evidence for the importance of microbial levan degradation in the plant context was found in the presence of 6-FEHs in non-fructan-accumulating species [85,86,87]. Levan of microbial origin is the only potential substrate for these enzymes. Recently, a chicory fructanase with the ability to degrade levan was studied, and the enzyme was shown to localize to the apoplast [88]. In conclusion, the action of apoplastic 6-FEHs, in concert with bacterial endo-levanases, may increase LOS production rates.

Within the tested levan-type fructans, no clear effect of branching was observed, fitting with the results on graminan and agavin. *B. subtilis* levan has a higher degree of branching compared to *H. smyrnensis* levan [94,118]. Fructan profiles of the concurrent LOS fractions verify this difference between both levans (Figure 2d). Priming effects were similar between both levan and LOS fractions. Nevertheless, priming with branched *B. subtilis* levan and LOS showed a marginal or significant increase in resistance, respectively. The absence of such effects for graminan and agavin may be explained by the fact that such structures are absent in bacteria, except for the lowest DP branched or neofructan by-products produced during the initial stages of fructansucrase reactions [73]. A function as potential MAMP is thus not very likely [81].

Sulfated *H. smyrnensis* levan was assayed as a priming agent. Although a significant disease reduction was found when taking into account lesion size distribution (Figure 4d), the LOS fraction obtained from sulfated levan performed better, similar to *B. subtilis* LOS. To our knowledge, this is the first time sulfated LOS have been produced in this way. We used an endo-levanase enzyme from *B. thetaiotaomicron*, previously shown to have high affinity for levan-type substrates. The enzyme showed no affinity towards inulin-type fructans. However, it was unknown whether this enzyme, or other levanases, can effectively degrade chemically modified levan, such as the sulfated levan used in this study. TLC results, as well as dry weight of low DP fructan after fractionation confirmed that this enzyme can produce sulfated LOS (Figure 3). It is important to note, however, that the sulfated form of LOS was not more effective in our priming assay compared to other LOS fractions.

When producing *H. smyrnensis* LOS, four different fractions were produced from the levan–levanase reaction mixture, fractionated using acetone precipitation (Figure 2c). Priming studies with these fractions clearly indicated a linear correlation between DP and priming efficacy. Rocket resistance to *B. cinerea* increased gradually when primed with lower average DP levan fractions, with a significant effect observed for fraction 3 and 4 (Figure 5c). The latter represents the LOS fraction subsequently used in all follow up experiments. On the other hand, clear effects for LOS priming were only observed when used at 5 g L^−1^. Perhaps fructan doses can be lowered in combination with other priming agents such as polyamines and GABA [52,53,56,81]. In addition, fructan seed or root priming may be more effective than leaf spraying and should be considered in future studies.

We show for the first time that effects of LOS priming persist in rocket after at least 7 d (Figure 5e). More detailed tests on priming duration will be needed when considering the use of fructans in agricultural applications. Priming effects were already observable for LOS after 24 h. Therefore, this reduced priming period was chosen for subsequent studies on carbohydrate metabolism and immune system parameters. A shorter time window may also minimize possible effects of changes in microbial community composition, which may also be affected by fructan priming, as described earlier. On the contrary, when *B. cinerea* inoculation is initiated sooner after priming, the fungus may benefit from altered apoplastic carbohydrate levels and/or residual priming residues.

LOS priming increased leaf total soluble sugars when compared to water and unprimed controls (Figure 6b). Overall, leaf detachment in itself had limited effects, but significant reduction of Glc, Fru and Suc was observed after inoculation with *B. cinerea* spores, especially in the control conditions. These reduced levels may be attributed to increased sugar consumption by the pathogen. Although Glc is the preferred carbon source, Fru can also be used. Suc cannot be taken up directly by *B. cinerea*; however this pathogen can produce sucrolytic enzymes [119]. In addition, pathogens often induce the activity of plant cwINVs to produce sufficient apoplastic Glc. *B. cinerea* was shown to influence cwINV inhibitors in order to increase cwINV activity [120]. Interestingly, total sugars were also significantly higher in LOS-primed leaves after infection, corresponding to results observed in *A. thaliana* [56]. Intracellular sugars are the biggest pool within the total sugar fraction. On the one hand, increases in intracellular sugars may indicate a decreased infection rate. On the other hand, increased intracellular sugars, as observed in LOS-primed leaves, can fuel the induction of defense responses by the cell [69]. LOS priming also affected starch content, but only after subsequent leaf detachment and infection (Figure 6c). Combined with small soluble sugar data, it is clear that LOS-primed leaves maintain a higher carbohydrate profile (soluble sugar and starch pools) both before and after infection.

Based on the ROS burst experiments, LOS priming itself did not lead to a clear induction of immune responses in rocket. This is perfectly in line with the priming concept, characterized by low energetic costs, preparing the cells for a future stress without an actual induction of defenses in the primed state [44,121]. On the other hand, a stronger response to infection is expected after priming. Our results indicate no clear induction of PAL after 48 h of infection, while myrosinase was induced in LOS- and sulfated LOS-primed leaves. On the contrary, PAL activity was induced by wounding effects during leaf detachment. The observed lower PAL activity after priming may be purely osmotic, since effects of HEG priming were similar to LOS priming (Figure 7a). In tobacco seedlings, on the other hand, inulin priming induced PAL gene expression [110] and both PAL and chitinase increased in kyoho grapes after *A. lappa* inulin priming [76]. It is possible that we missed the time window in which PAL activity increased due to priming. Earlier studies have indicated a fast PAL induction with chitosan as priming agent, showing a biphasic induction within the first 18 h [122]. The decreased PAL activity after infection, although on the edge of statistical significance, as compared to IC may indicate that *B. cinerea* actively suppresses plant PAL activity. Intriguingly, hijacking PAL of the plant has been observed for another necrotrophic pathogen, *Ascochyta rabiei,* through a secreted effector [123]. However, it is unclear whether similar mechanisms are in place in the case of *B. cinerea,* warranting further studies. Myrosinase levels were also induced mainly by wounding effects. The efficacy of glucosinolate-breakdown products against *B. cinerea* is limited, since this necrotroph harbors a specific efflux transporter to reduce toxic effects [124].

Janse van Rensburg et al. [56] showed that fructans themselves could not induce a ROS burst in *A. thaliana*. The same was observed for rocket, confirming that fructan species act as genuine primers and not as defense elicitors. As expected, flg22 produced a significantly higher ROS burst [125] compared to H_2_O and unmodified fructans (Figure 7b,c). Sulfated LOS on the other hand produced a burst significantly higher than all treatments except flg22, indicating a possible immediate signaling after sulfated LOS priming through ROS. It will be interesting to compare sulfated LOS and flg22 in equimolar concentrations and compare the observed bursts. Sulfated levan already showed interesting effects in animal systems [79,126] and a direct induction of plant immunity has already been observed for other sulfated carbohydrates, such as algal ulvans, fucans and laminarins [127,128]. Generally, it will also be interesting to study fructans as direct elicitors on fructan-primed plants instead of unprimed ones, as this will give more insight into the effect of multiple fructan priming events, which should be considered for potential future applications in pest management.

As discussed earlier, apoplastic sugar dynamics are important in plant-microbe interactions, including the interaction with plant pathogens. Generally, apoplastic sugars decreased significantly after infection, compared to primed conditions, showing similar trends as observed for sugars in full leaf extracts (Figure 8g). Total sugar levels were not higher after infection in LOS-treated leaves compared to H_2_O controls. However, in this case HEG priming may be considered a better control condition. Apoplast sugar levels, and especially Suc, were high for H_2_O-treated leaves after priming (Figure 8e). These levels differ clearly from unprimed and HEG conditions and may indicate exposure of the leaves to a hypo-osmotic shock, caused by leaf water spraying. This effect may trigger the activation of SWEET sugar transporters, increasing the apoplastic concentrations of Suc to reverse the hypo-osmotic conditions. Accordingly, also cwINV activity was visibly higher after H_2_O priming (Figure 6d), which may also be linked to hypo-osmotic stress conditions. Compared to the HEG control, LOS and sulfated LOS showed clearly higher apoplastic sugar levels, with significant differences for both Fru and Suc (Figure 8d,e), indicating that LOS priming may activate SWEET sugar transporters as well, temporarily increasing apoplast sugar levels.

When investigating apoplastic dynamics in more detail for LOS priming, the time point at 24 h after priming was marked by a clear increase in all small soluble sugars (Figure 9), and levels declined significantly at later time points in LOS-treated samples. Our results show that LOS priming influences sugar dynamics between the in- and outside of the cell, urging further research on how LOS signals during priming affect SWEETs and other sugar transporters. The developed AF extraction method showed minimal contamination and allowed a strong starting point to undertake such studies in the future. The observed temporal higher apoplastic sugar concentrations after 24 h LOS priming may trigger RGS1 (sugar specific) or even MAPK signaling pathways (any osmolyte) since 25 mM mannitol already activated MPK3 and MPK6 [129,130], and this represents an exciting new avenue of research. After infection of LOS-primed leaves, starch content was also significantly higher (Figure 6c). cwINV activity was not increased under these conditions, even though Suc/hexose ratios of total leaf extracts were significantly different after infection (Figure S4d; ratios in AF were similar to control conditions). The observed effects fit with the sweet immunity concept [8,69]. In conclusion, our results indicated effects of LOS priming on carbohydrate dynamics in the leaf. Priming itself induced higher hexose and total sugar levels compared to control conditions in terms of total leaf sugars (Figure 6b). Their increase likely led to the observed increased apoplastic levels of Glc, Fru and Suc (Figure 8d,e and Figure 9a).

After subsequent infection with *B. cinerea*, higher concentrations of Suc and hexoses were also observed in total leaf extracts of LOS-primed samples. However, this increase was linked to intracellular sugar levels, since apoplastic levels were similar to those of IC conditions, indicating that the pathogen preferentially consumed sugars from the apoplastic continuum (Figure 8). For this purpose, *B. cinerea* can hijack SWEET transporters of the plant to increase sugar efflux from the host cell, as observed in studies on *A. thaliana* and *Vitis vinifera* [131,132,133].

Finally, we confirmed that the leaf-sprayed LOS actually reached the apoplastic space. Our results clearly indicated that at least part of the sprayed LOS could reach the apoplast, as observed already 1 h after priming (Figure 9e). At later time points, however, we observed a significant drop in apoplastic LOS. No LOS transporters or other uptake systems have previously been discovered in plants, while this is possible for microbes, as demonstrated in a recent study on *B. thetaiotaomicron* [134]. In the case of FOS, transporters have been found for several bacterial species with specificity towards the lowest DP FOS [112,135,136]. Although the uptake of LOS by either endophytes or plant cells cannot be completely dismissed without further research, the observed decrease in LOS most likely involves degradation by apoplastic fructanases, either of plant or microbial origin. If LOS is indeed sensed as a priming signal, our results indicate that concentrations gradually decline to prevent continuous sensing. Although FOS priming also led to similar sugar dynamics in the apoplast (Figure 10), the inefficiency of disease priming can most probably be explained by the fact that *B. cinerea* harbors inulinase activity [114], indicating it can use FOS as a food source, further confirmed by our own analysis (Figure 10c). This is different from the case of LOS priming, since fungal fructanases have no affinity towards levan-type substrates [137]. Additionally, in the case of the *Venturia*/apple pathosystem, inulin proved inefficient in priming as compared to levan, and this could be linked to the fact that the fungus was able to grow on inulin but not on levan [78].

## 5. Conclusions

In this manuscript, the efficacy of fructan priming on leaves of *E. sativa* challenged with *B. cinerea* was investigated. Our data indicated increased pathogen resistance after priming with LOS, independent of its origin (*H. smyrnensis*, *B. subtilis* or sulfated LOS). The results regarding inulin priming differ from previous studies in *L. sativa* and *A. thaliana*, showing no significant increase in disease resistance. Testing different DP levan fractions, we confirmed the increased priming efficiency with lowering average DP. Sulfated LOS directly stimulated ROS burst induction in unprimed leaves. *H. smyrnensis* LOS induced no significant ROS burst, indicating a priming effect, rather than a direct immune-eliciting effect. In terms of sugar dynamics, LOS priming increased total sugar levels both after priming and subsequent infection, and similar effects were observed for starch. An apoplastic increase in Glc, Fru and Suc was also observed after LOS priming. These data indicate potential apoplastic sugar signaling pathways in response to LOS priming, as well as a different metabolic status of LOS-primed leaves at the time of infection. This is the first paper describing carbohydrate priming in rocket, testing a large array of different fructan types. For the first time, the hydrolysis of sulfated levan by an endo-levanase has been performed, and sulfated levan and LOS were tested in the plant immunity context. Finally, the observed LOS-dependent changes in apoplast and intracellular sugars provide an interesting starting point to study LOS priming effects in more detail in the future. Future research should also further explore immunity and resistance markers (related to phytohormones and main immune system pathways) in light of fructan priming.

## Data Availability

All data supporting the findings of this study are available within the paper and its Supplementary Materials published online.

## Supplementary Materials

The following supporting information can be downloaded at: https://www.mdpi.com/article/10.3390/biom12030370/s1, Figure S1: Heterologous expression of *Bacteroides thetaiotaomicron* endo-levanase and substrate specificity, Figure S2: HPAEC-IPAD profile of *Triticum aestivum* graminan and *Agave tequilana* agavin using as a priming agent on rocket leaves, Figure S3: Purification of selected fructan solutions to remove high levels of small soluble sugars, Figure S4: Effect of fructan priming in the rocket-*Botrytis cinerea* pathosystem on small soluble sugars and vINV activity, Figure S5: Effect of fructan priming on myrosinase activity in the rocket-*Botrytis cinerea* pathosystem, Figure S6: Effect of fructan priming on apoplast small soluble sugar levels in the rocket-*Botrytis cinerea* pathosystem.
