# Supplementary material for: Sweet Immunity Aspects during Levan Oligosaccharide-Mediated Priming in Rocket against Botrytis cinerea
Source: Biomolecules. 2022 Feb 25;12(3):370. doi: 10.3390/biom12030370 (PMC8945012; doi:10.3390/biom12030370)
Supplement: Supplementary file 1 [file biomolecules-12-00370-s001.zip › biomolecules-1560215-supplementary.pdf]

biomolecules

MDPI

Supplementary Materials

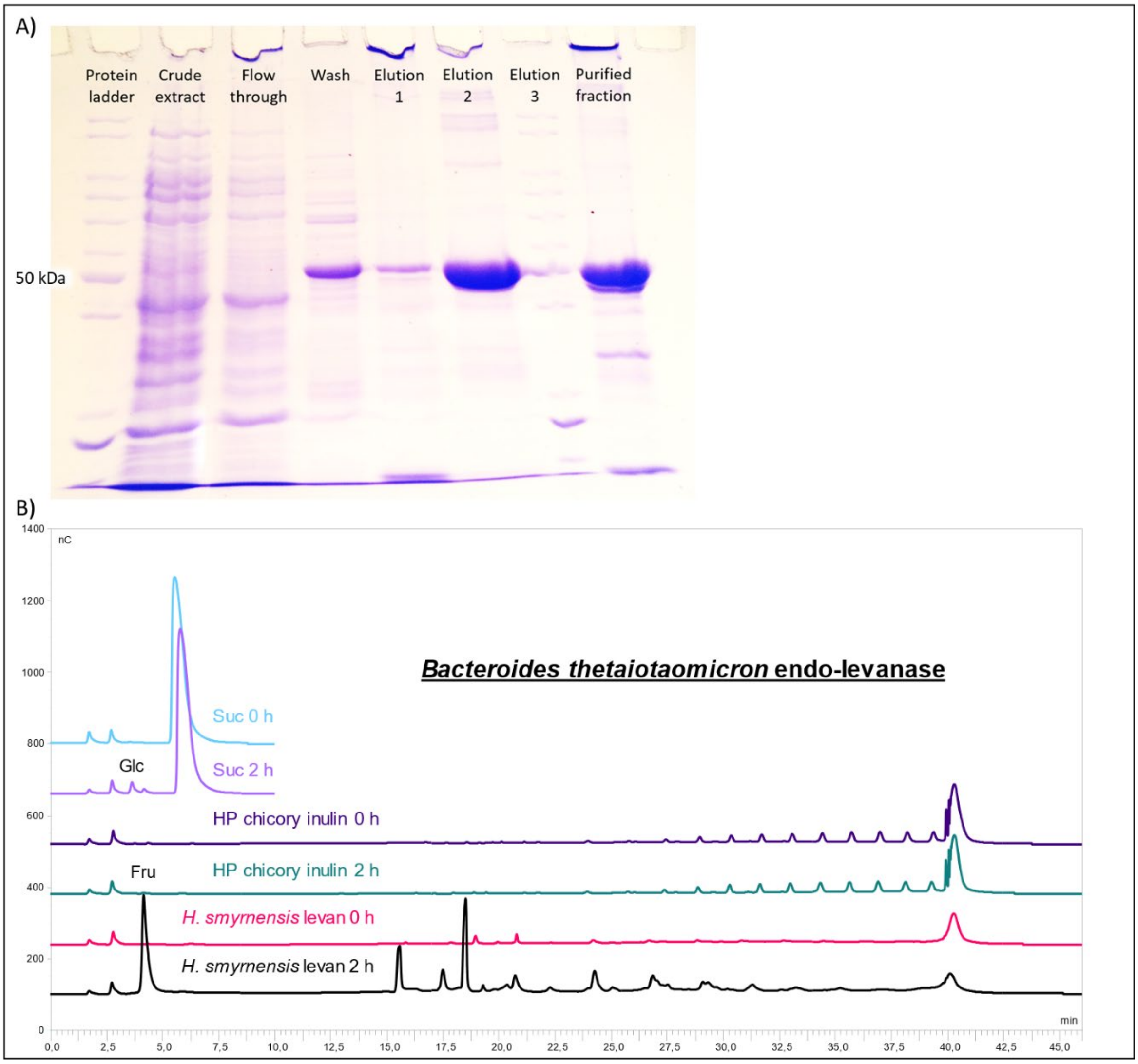

Figure S1. Heterologous expression of *Bacteroides thetaiotaomicron* endo-levanase and substrate specificity. (**A**) SDS-PAGE shows a clear band for the expressed protein in the elution fraction and after further purification. The observed Mw corresponds to the estimated size of 57 kDa. (**B**) The enzyme was incubated with different substrates to test substrate specificity: Suc, HP chicory inulin and *Halomonas smyrnensis* levan. X-axis depicts retention time (min), while y-axis indicates amperometric signal (nC).

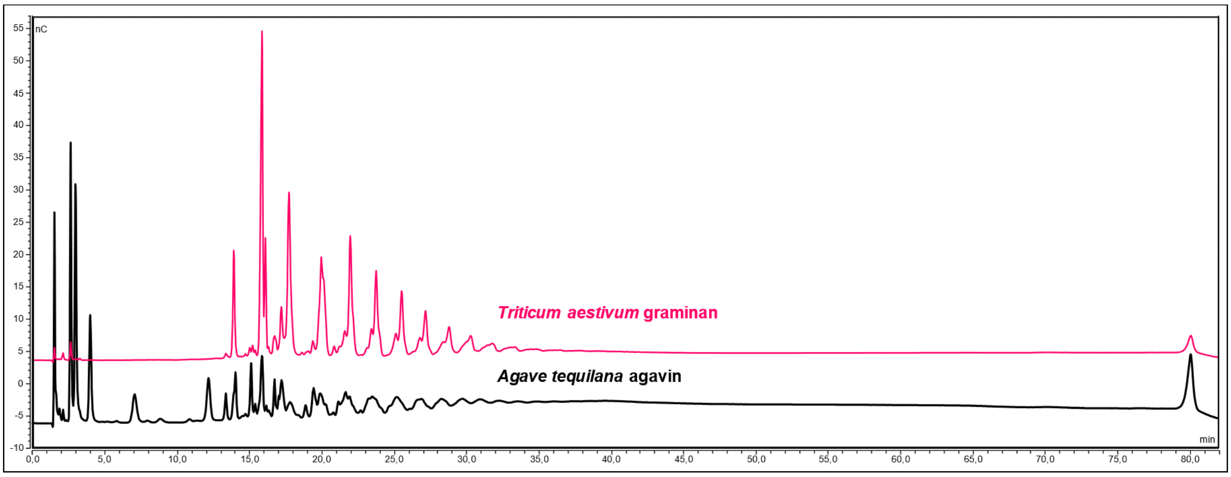

Figure S2. HPAEC-IPAD profile of *Triticum aestivum* graminan and *Agave tequilana* agavin used as a priming agent on rocket leaves. Fructans containing high amounts of small soluble sugar contamination (elution time 2-4 min) were further purified before use. X-axis depicts retention time (min), while y-axis shows the amperometric signal (nC).

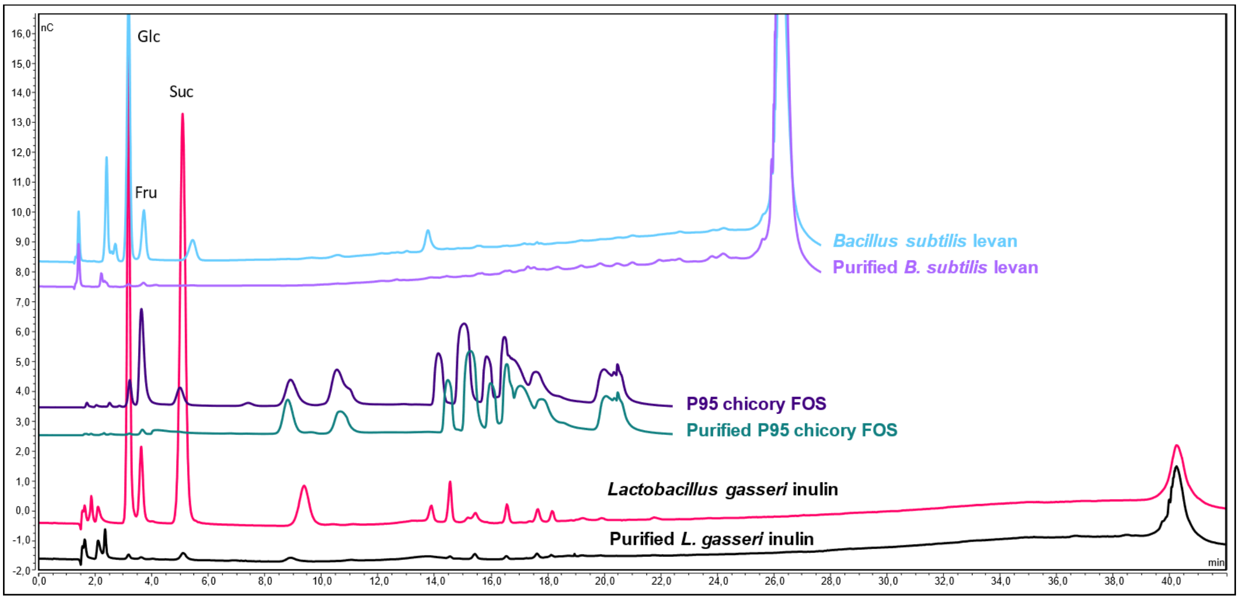

Figure S3. Purification of selected fructan solutions to remove high levels of small soluble sugars. Purifications were performed on a NH2 (amino) SPE sorbent column with acetonitrile. X-axis shows retention time (min) and y-axis indicates amperometric signal (nC).

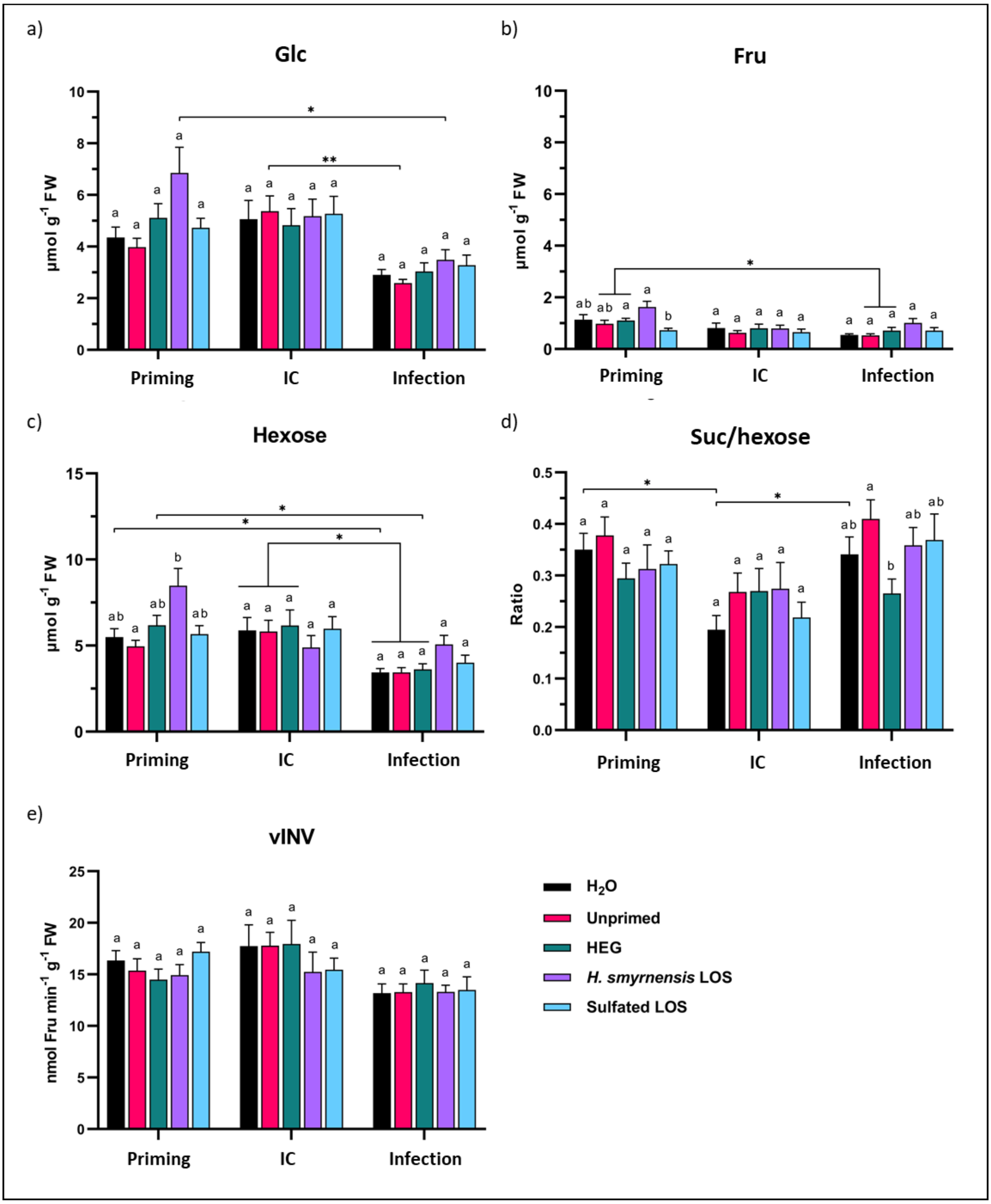

Figure S4. Effect of fructan priming in the rocket-*Botrytis cinerea* pathosystem on small soluble sugars and vINV activity. Leaf samples were taken from primed or infected leaves. Infection control leaves (IC) incubated only with infection buffer where also sampled. (**a**) Glucose (Glc) and (**b**) fructose (Fru) levels were measured on HPAEC-IPAD. (**c**) Total hexose levels and sucrose (**d**) (Suc)/hexose ratios were calculated based on these data. (**e**) vINV activity was measured on HPAEC-IPAD through Fru production. Bars represent the mean ± SEM. Six biological replicates were used per treatment. The experiment was repeated 3 times with consistent results. Letters indicate significant differences between treatments within the same time point ($p < 0.05$). Asterisks indicate significant differences of the same treatment between time points (* $p < 0.05$).

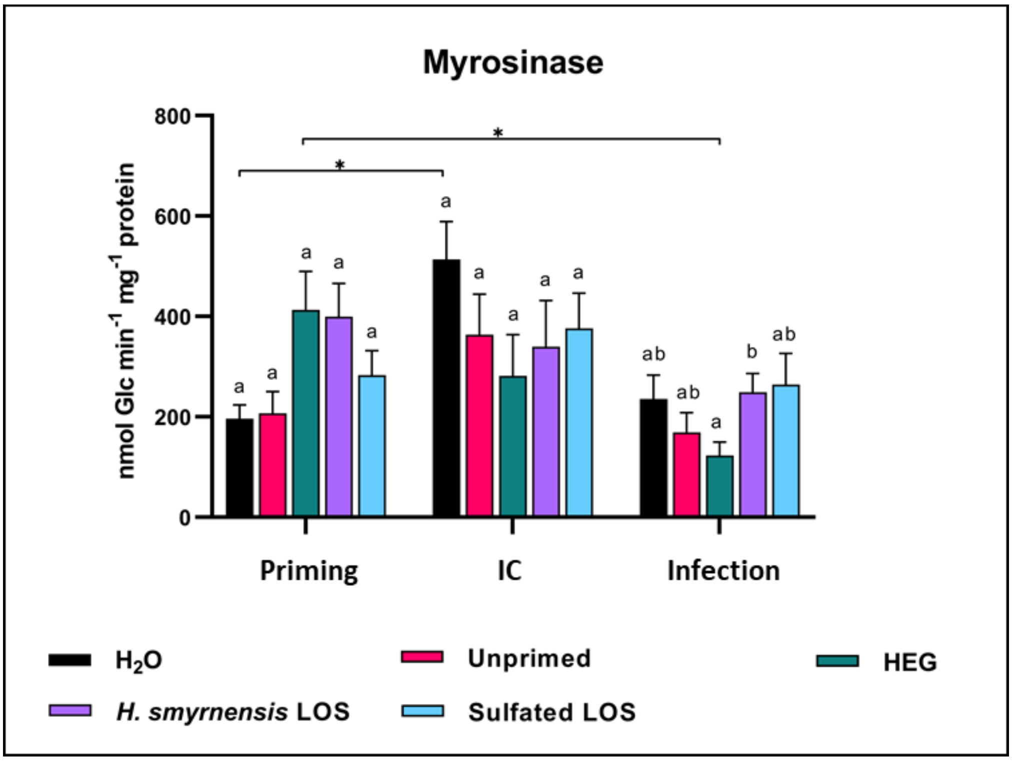

Figure S5. Effect of fructan priming on myrosinase activity in the rocket-*Botrytis cinerea* pathosystem. Samples were taken after priming, from infected leaves and infection control (IC) treated leaves. Myrosinase activity was analysed through incubation with a glucosinolate, measuring the production of Glc on HPAEC-IPAD. Bars represent the mean ± SEM. Six biological replicates were used per treatment. The experiment was repeated 2 times with consistent results. Letters indicate significant differences between treatments within the same time point ($p < 0.05$). Asterisks indicate significant differences of the same treatment between time points (* $p < 0.05$).

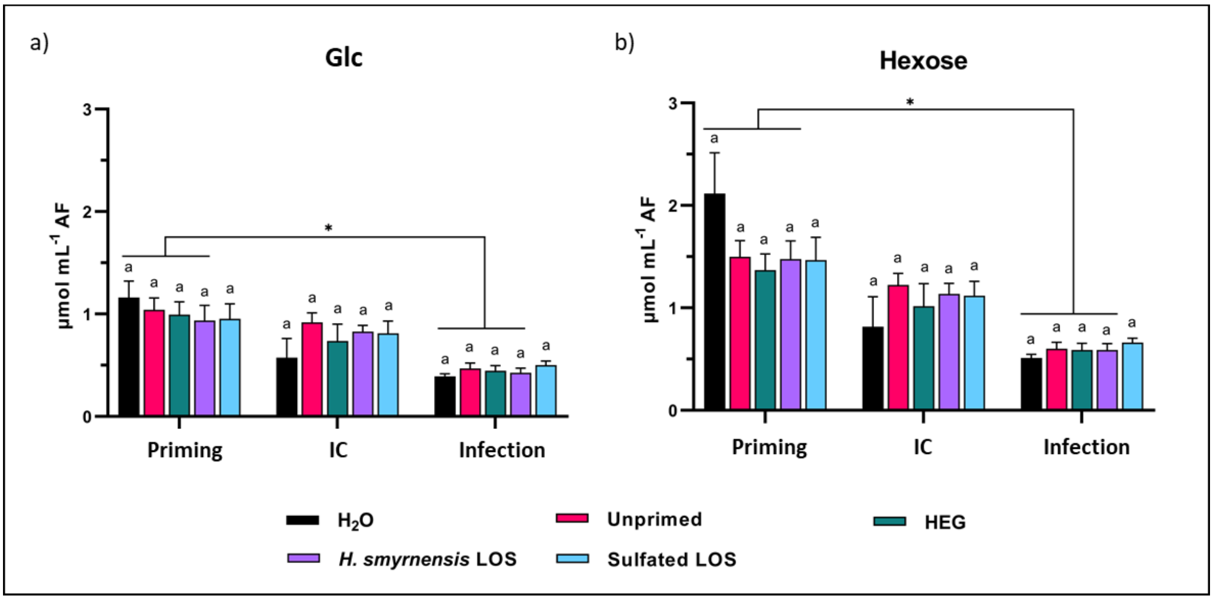

Figure S6. Effect of fructan priming on apoplast small soluble sugar levels in the rocket-*Botrytis cinerea* pathosystem. Apoplastic fluid (AF) was extracted from leaves after priming or infection. Infection control (IC) samples were also taken. (**a**) Glucose (Glc) was measured on HPAEC-IPAD. (**b**) Total hexose levels were calculated from Glc and Fru data. Bars represent the mean ± SEM. Six biological replicates were used per treatment. Letters indicate significant differences between treatments within the same time point ($p < 0.05$). Asterisks indicate significant differences of the same treatment between time points (* $p < 0.05$).
